# Theoretical Investigations on the Reactivity of Hydrogen Peroxide toward 2,3,7,8-Tetrachlorodibenzo-*p*-dioxin

**DOI:** 10.3390/molecules23112826

**Published:** 2018-10-31

**Authors:** Weihua Wang, Yuhua Wang, Wenling Feng, Wenliang Wang, Ping Li

**Affiliations:** Key Laboratory of Life-Organic Analysis, School of Chemistry and Chemical Engineering, Qufu Normal University, Qufu 273165, China; wyhqfnu@163.com (Y.W.); wlfengqf@163.com (W.F.); wlwangqf@126.com (W.W.)

**Keywords:** 2,3,7,8-tetrachlorodibenzo-*p*-dioxin, hydrogen peroxide, reaction mechanisms, theoretical calculations

## Abstract

Acquiring full knowledge of the reactivity of 2,3,7,8-tetrachlorodibenzo-*p*-dioxin (TCDD) is crucial for the better understanding of the transformation and degradation of TCDD-like dioxins in the environment. To clarify the reactivity of the organic hydroperoxides toward TCDD, in this study, the reactions between the neutral/anion of the hydrogen peroxide (H_2_O_2_) and TCDD have been systematically investigated theoretically. It was found that the neutral H_2_O_2_ is relatively difficult to react with TCDD compared with its anion, exhibiting the pH dependence of the title reaction. As for the anion of H_2_O_2_, it reacts with TCDD through two reaction mechanisms, i.e., nucleophilic substitution and nucleophilic addition. For the former, the terminal O atom of HO_2_^−^ nucleophilically attacks the C atom of the C-Cl bond in TCDD to form an intermediate containing an O-O bond, accompanying the dissociation of the chlorine atom. For the latter, the terminal O atom of HO_2_^−^ can be easily attached to the C atom of the C-O bond in TCDD, resulting in the decomposition of C-O bond and the formation of an intermediate containing an O-O bond. For these formed intermediates in both reaction mechanisms, their O-O bonds can be homolytically cleaved to produce different radicals. In addition, the selected substitution effects including F-, Br-, and CH_3_- substituents on the above reactions have also been studied. Hopefully, the present results can provide new insights into the reactivity of the organic hydroperoxides toward TCDD-like environmental pollutants.

## 1. Introduction

It is well known that persistent organic pollutants (POPs) are organic compounds that persist in the environment for long periods of time, which have been regulated under the Stockholm Convention. As one of the typical POPs, polychlorinated dibenzo-*p*-dioxins (PCDDs) are a class of environmental pollutants arising from anthropogenic sources (e.g., waste incineration of chlorine-containing substances) and natural sources (e.g., volcanoes and forest fires). They have imposed many adverse impacts on ecological environments and human health. Especially, as the most toxic dioxin, 2,3,7,8-tetrachlorodibenzo-*p*-dioxin (TCDD) has been classified as a Group 1 carcinogen by the International Agency for Research on Cancer (IARC). To understand the formation [1,2,3,4,5,6,7,8] and transformation or degradation [9,10,11,12,13,14,15,16,17,18,19,20,21,22,23,24,25,26,27] mechanisms of PCDDs, many experimental and theoretical investigations have been carried out. Several methods involving microbial degradation [9,10,11], photocatalysis [12,13,14], incineration and thermal treatment [15,16], have been proposed to degrade PCDDs. However, the high stabilities of PCDDs make them difficult to react with other species due to their special structures. In fact, only a few free radicals, such as OH, NO_3_, and O_3_, can react with PCDDs. Therefore, seeking new reactive species that can react with PCDDs is crucial for the study of the transformation of PCDDs.

Recently, more and more studies have shown experimentally that hydrogen peroxide (H_2_O_2_) and its derivatives can react with chlorinated benzoquinones under mild conditions, producing highly reactive free radicals (e.g., hydroxyl radical, organic alkoxyl radicals, and quinone ketoxy radicals) [28,29,30,31]. Moreover, these produced radicals can cause oxidative damages to DNA, protein, and lipids [32]. In addition, it was found that explicit water molecules play an important role in the formation of these free radicals theoretically [33,34].

Inspired by the abovementioned high reactivity of H_2_O_2_ toward chlorinated benzoquinones [28,29,30,31], we wonder if H_2_O_2_ can react with TCDD since chlorinated benzoquinones and TCDD possess some similarities in structure and property, where both of them have a planar ring structure containing C-Cl bond and are good electron acceptors [35,36,37,38]. If they do react, what are the detailed reaction mechanisms? Obviously, clarification of above questions can enable us to better understand the potential reactivity of H_2_O_2_ and its derivatives in the transformation of TCDD. Unfortunately, to the best of our knowledge, no relevant studies have been reported so far. In view of the high toxicity of TCDD, theoretical studies on the above reaction are highly desirable.

Therefore, in this study, using TCDD as a model compound for PCDDs, its reactions with neutral H_2_O_2_ and its anion have been systematically explored using the density functional theory (DFT). The possible reaction mechanisms have been discussed for the whole reaction. Moreover, the substitution effects of F-, Br- and CH_3_- groups on the title reaction have also been explored. Expectedly, the present results not only enable us to better understand the potential reactivity of organic hydroperoxides, but also promote the performance of the related experimental studies on the transformation of the TCDD-like environmental pollutants.

## 2. Results and Discussion

In view of the fact that neutral H_2_O_2_ and its anion coexist due to the occurrence of the acid-base dissociation equilibrium in solution, the reactions of the neutral and anion of H_2_O_2_ with TCDD are discussed respectively.

### 2.1. Reaction of Neutral H_2_O_2_ with TCDD

Similar to the reaction between chlorinated benzoquinones and H_2_O_2_ [33,34], the reaction between H_2_O_2_ and TCDD is initiated by the formation of an initial intermediate. Then, nucleophilic attack of H_2_O_2_ on TCDD occurs to produce an unstable intermediate containing an O-O bond. Finally, the unstable intermediate decomposes via the homolytical cleavage of the O-O bond, resulting in the production of radicals. The detailed reaction mechanisms are described as follows.

#### 2.1.1. Direct Reaction of the Neutral H_2_O_2_ with TCDD

**Figure 1:**
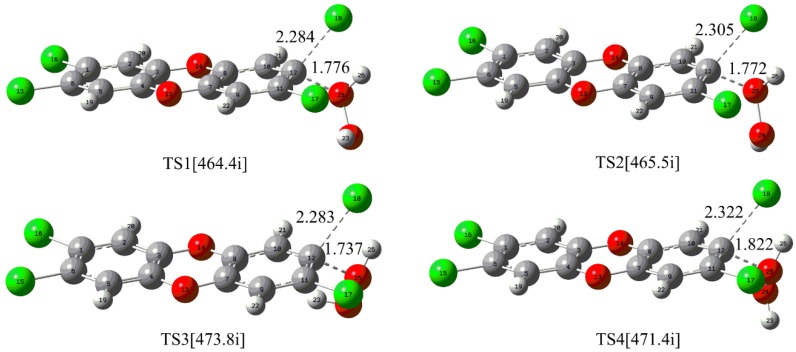
Calculated transition states for the direct nucleophilic attack of neutral H_2_O_2_ on TCDD. The selected distances are given in Å and the data in square brackets refer to the imaginary frequency of the transition state, which is true for the remaining figures.

For the direct reaction of the neutral H_2_O_2_ with TCDD, four nucleophilic attack modes named as 1, 2, 3, and 4 have been constructed considering the D_2h_ symmetry of TCDD and the different orientations of the H atom in H_2_O_2_. As shown in Figure 1, the corresponding transition states TSn (n = 1–4) for each mode have been located.

As displayed in Figure 1, the O atom of H_2_O_2_ nucleophilically attacks the C atom of the C-Cl bond in TCDD, accompanying the proton transfer (PT) from the attacking O atom of H_2_O_2_ to the dissociated chlorine atom of TCDD. As presented in Table 1, the free energy barriers for the four nucleophilic attack modes are 62.43, 62.39, 64.04 and 61.63 kcal/mol, respectively. Similarly, the M06-2X/6-311++G(d,p) level of theory can also give consistent results. Obviously, such high energy barriers suggest that it is very difficult for the title reaction to occur under normal conditions.

Can the bulk solvent effects promote the above processes? To clarify this point, the solvent effects in aqueous solution have been considered employing the SMD model. As presented in Table 1, the free energy barriers mentioned above have been changed slightly upon solvation, where the changes of the free energy barriers range from 1.20 to −1.07 kcal/mol. Therefore, it is still difficult for the direct reaction between neutral H_2_O_2_ and TCDD to take place even though solvent effects are included.

#### 2.1.2. Water-Assisted Reaction of Neutral H_2_O_2_ with TCDD

In view of the fact that a water molecule plays an important catalytic role in promoting the PT process and the above NAP involves the PT process, the reaction of H_2_O_2_ with TCDD has been explored below with the assistance of different numbers of explicit water molecules ranging from one to three on the basis of the above nucleophilic attack mode 1. For simplicity, the symbols IMx(nw) and TSx(nw) have been employed to stand for the optimized intermediates (IMs) and transition states (TSs), where x and n denote the formation sequence of the mentioned species and the numbers of water molecules involved, respectively. For instance, IM1(1w) and IM2(2w) represent the first and second intermediates in the reaction pathways involving one and two water molecules, respectively. For the four reaction pathways involving zero, one, two, and three water molecules, they have been named as pathways A, B, C and D, respectively.

##### Formation of the Initial Intermediate

**Figure 2:**
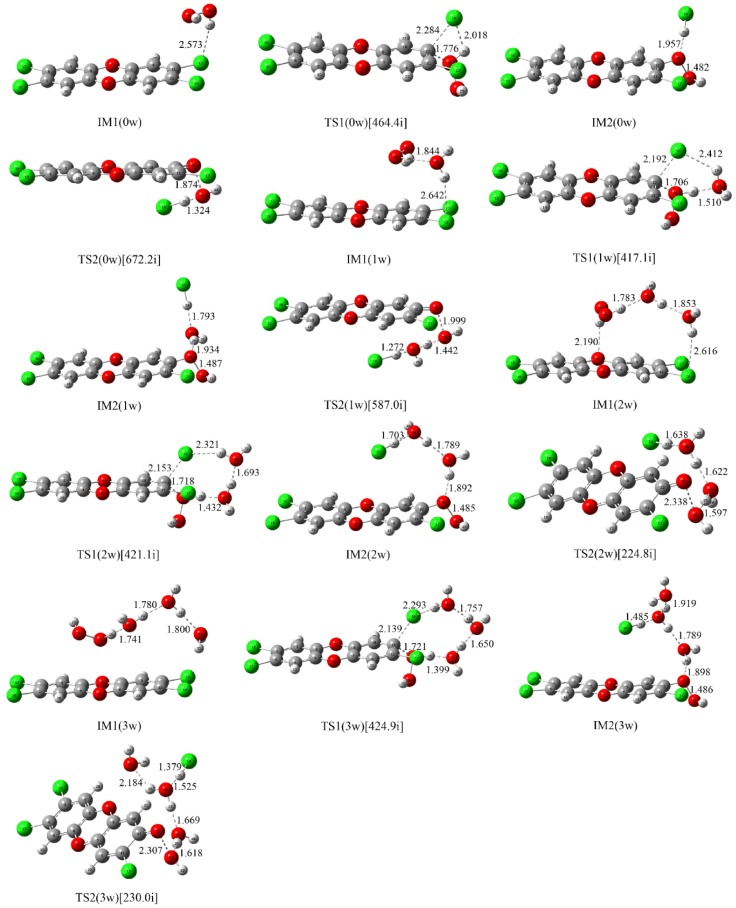
Optimized intermediates (IM) and transition states (TS) in the available reaction pathways of the reaction between neutral H_2_O_2_ and TCDD.

As the first step of the whole reaction, the initial intermediates have been explored based on the IRC analyses of the corresponding transition states TS1(nw)_n=0–3_ in the NAPs. As shown in Figure 2, different initial intermediates IM1(nw)_n=0–3_ have been located. Correspondingly, the molecular graphs and topological analyses of them are given in Figure S1 and Table S1 of the Supplementary Materials, respectively.

As displayed in Figure 2 and Figure S1, TCDD, H_2_O_2_, and H_2_O interact with each other via intermolecular H-bonds in the formed IMs, which can be confirmed by the presence of the corresponding BCPs. In IM1(0w), one of the H atoms of H_2_O_2_ forms an intermolecular H-bond with the chlorine atom of TCDD. For the water molecules introduced, they interact with both TCDD and H_2_O_2_ simultaneously via intermolecular H-bonds. As presented in Table S1, the above intermolecular H-bonds are mostly predominated by the electrostatic interactions as can be seen from the positive values of the ∇^2^*ρ*_bcp_ and *H*_bcp_ of the electron density at the BCPs. Actually, this point is also be reflected by the large H-bonding distances shown in Figure 2.

As presented in Table 2, IM1(0w) has been stabilized by 1.52 kcal/mol relative to those of the separated reactants. Moreover, the stabilization energy increases with the increasing of the numbers of water molecules. For example, IM1(1w), IM1(2w), and IM1(3w) have been stabilized by about 6.83, 13.82 and 21.31 kcal/mol, respectively. Meanwhile, the enthalpy changes for these formation processes are negative values, suggesting that the formation processes of these IMs are exothermic reactions. Importantly, the released reaction heat increases with the increasing of the numbers of water molecules, which is necessary for the following NAP.

##### Nucleophilic Substitution Process

**Figure 3:**
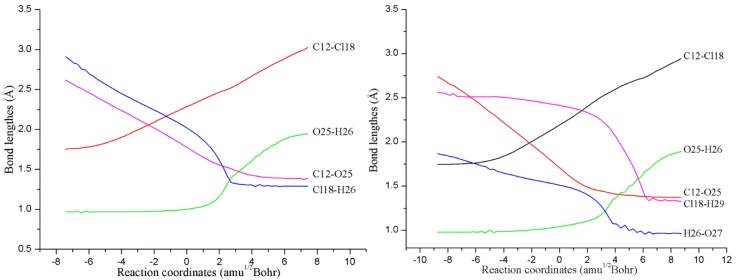
Variations of the selected bond lengths along with the IRC of the transition states TS1(0w) (**left**) and TS1(1w) (**right**).

As displayed in Figure 2, all the transition states TS1(nw)_n=0–3_ in the NAPs have been located, which have been further verified by the IRC calculations. As shown in Figure 3, the microscopic details during the NAP can be observed. For example, for the direct reaction in the absence of water molecules, one of the O atoms (O25) of H_2_O_2_ directly attacks the C atom (C12) of TCDD, accompanying the simultaneous dissociation of the H26 atom of H_2_O_2_ and Cl18 atom of TCDD. As a result, the second intermediate IM2(0w) containing an O-O bond can be formed. Note that the O25-H26 bond of H_2_O_2_ begins to increase significantly until the formation of the transition state, exhibiting the asynchrony of the nucleophilic substitution reaction. As for the process assisted by one water molecule, as shown in Figure 3, the introduced water molecule accepts the proton of H_2_O_2_ and donates its own proton to the chlorine atom of TCDD simultaneously, reflecting the bridge role of water molecule in the assistance of PT. Moreover, no zwitterionic species have been located during the PT process. Therefore, the above PT processes should proceed concertedly. Similarly, the same is also true for the processes involving two and three water molecules.

To better understand the catalytic role of water molecules in the above process, the selected distances involving the attacked C atom of TCDD in TSs have been analyzed. As displayed in Figure 2, the distances between the dissociated Cl atoms and its linked C atom (R_C···Cl_) in TS1(nw)_n=0–3_ decrease with the increasing of the numbers of water molecules. For instance, R_C···Cl_ is 2.284, 2.192, 2.153 and 2.139 Å in TS1(nw)_n=0–3_ involving zero, one, two, and three water molecules, respectively. On the other hand, the opposite is true for the distance between the attacking O atom of H_2_O_2_ and the attacked C atom of TCDD. Obviously, less structural deformations occur for H_2_O_2_ and TCDD in the presence of water molecules compared with the direct reaction, implying the decrease of the energy barriers with the assistance of water molecules.

Expectedly, as shown in Table 2, the original electronic energy barrier in the NAP has been decreased significantly with the increasing of the numbers of water molecules. For example, it has been decreased by 28.38 to 23.72 kcal/mol with the assistance of three water molecules, exhibiting the positive catalytic role of water molecules. On the other hand, the free energy barriers have been changed slightly.

##### Cleavage of the O-O Bond

After the NAP, the second intermediates IM2(nw)_n=0–3_ containing an O-O bond have been formed. As shown in Figure 2, all of them have been characterized by the intermolecular H-bonds, which can be further confirmed by the presence of the BCPs as shown in Figure S1.

As shown in Figure 2, the O-O bonds in IM2(nw)_n=0–3_ have been elongated more or less compared to that of the H_2_O_2_, indicating the weakening of them. Actually, as presented in Table S1, this point can be further confirmed by the decreases of the electron density at the BCP of the O-O bonds upon the formation of IM2(nw)_n=0–3_. Moreover, to further evaluate the strength of the O-O bonds, the vertical and adiabatic bond dissociation energies (BDEs) of the O-O bonds have been calculated for IM2(nw)_n=0–3_ as well as that of H_2_O_2_ for comparison. Here, the vertical BDE is calculated as the energy difference between the optimized intermediate and the dissociated fragments without considering the structural relaxation. As for the adiabatic BDE, it is calculated as the enthalpy difference between the optimized species before and after dissociation. As presented in Table 3, the O-O bond has been significantly weakened upon the formation of IM2(nw)_n=0–3_. For example, the vertical BDE of the O-O bond of H_2_O_2_ (48.62 kcal/mol) has been decreased by about 28.30 to 20.32 kcal/mol in IM2(0w). Meanwhile, significant decreases for the adiabatic BDE have also been observed. Therefore, it is easy to homolytically cleave these O-O bonds in IM2(nw)_n=0–3_ thermodynamically.

Moreover, as shown in Figure 2, the corresponding TSs for the cleavage of the O-O bond have also been located. As presented in Table 2, for IM2(0w), the calculated free energy barrier is 6.27 kcal/mol relative to IM2(0w). Moreover, the energy barriers are 8.53, 14.09 and 16.37 kcal/mol in the presence of one, two, and three water molecules, respectively. In fact, these transition states are lower in energy relative to the initial reactants. For example, TS2(2w) and TS2(3w) are lower in energy by 9.04 and 12.01 kcal/mol than the initial reactants. Thermodynamically, the calculated Gibbs free energy changes in the conversion process from IM2(nw)_n=0–3_ to products are −3.42, −4.40, −3.87, and −2.77 kcal/mol for the pathways A, B, C, and D, corresponding to the equilibrium constants of 3.22 × 10^2^, 1.69 × 10^3^, 6.82 × 10^2^, and 1.07 × 10^2^, respectively. Therefore, it is feasible for the intermediates IM2(nw)_n=0–3_ to produce two radicals via the homolysis of the O-O bond.

Compared with the NAPs, as shown in Table 2, the energy barriers for the cleavage of the O-O bond are relatively small. Therefore, the NAP should be the rate-determining step for the above reaction.

### 2.2. Reaction of TCDD with HO_2_^−^ Anion

Moreover, the reaction of the anion of H_2_O_2_ with TCDD has also been explored. As a result, two reaction mechanisms, i.e., nucleophilic substitution and nucleophilic addition, have been verified below.

#### 2.2.1. Nucleophilic Substitution Process

**Figure 4:**
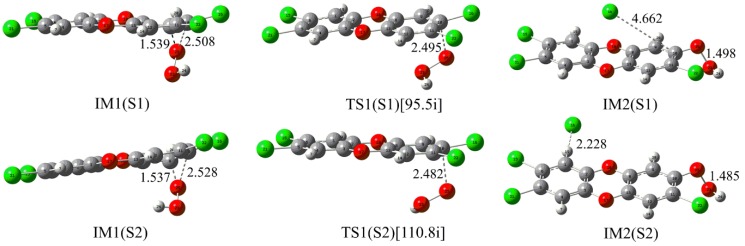
Optimized IMs and TSs in the nucleophilic substitution reaction of HO_2_^−^ with TCDD.

As shown in Figure 4, two different nucleophilic attack modes, i.e., modes S1 and S2, have been designed based on the structural symmetry of TCDD. Similar to the above reaction involving neutral H_2_O_2_, two initial intermediates IM1(S1) and IM1(S2) for the two modes have first been located. Subsequently, the nucleophilic attack of HO_2_^−^ on TCDD occurs via transition states TS1(S1) and TS1(S2), leading to the production of the second intermediates IM2(S1) and IM2(S2) containing an O-O bond. As expected, the following process should proceed to produce radicals via the homolytic cleavage of the O-O bond. Especially, unlike the reaction of the neutral H_2_O_2_ mentioned above, no explicit water molecules are required here.

Further energy analyses suggest that the two initially formed intermediates IM1(S1) and IM1(S2) have been stabilized by about 24.16 and 23.17 kcal/mol relative to the initial reactants, respectively. Similarly, the corresponding transition states are also lower in energy by about 16.08 and 15.39 kcal/mol relative to the initial reactants. As a result, the calculated free energy barriers for the nucleophilic attack of TCDD by HO_2_^−^ are 7.64 and 7.08 kcal/mol relative to the initial intermediates, respectively. In aqueous solution, the corresponding free energy barriers are 23.64 and 23.03 kcal/mol relative to the initial intermediates, respectively. Compared with the results in the gas phase, the important solvent effects should be stressed here.

Overall, for the nucleophilic substitution process, the reaction of HO_2_^−^ with TCDD is more favorable than that of the reaction involving the neutral H_2_O_2_, exhibiting the dependence of the title reaction on the pH value of the media.

#### 2.2.2. Nucleophilic Addition Process

**Figure 5:**
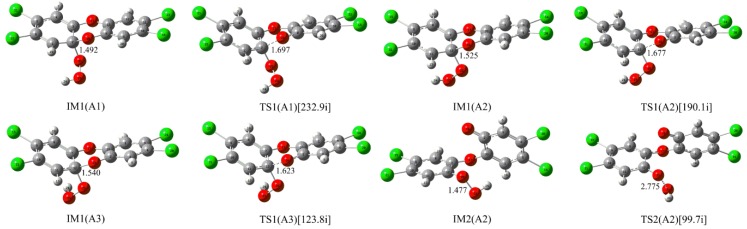
Optimized IMs and TSs in the nucleophilic addition reaction of HO_2_^−^ with TCDD.

Besides the nucleophilic substitution reaction, the nucleophilic addition reaction of HO_2_^−^ has also been observed. As shown in Figure 5, the terminal O atom of HO_2_^−^ can be attached to the C atom of the C-O bond in TCDD through three addition modes (A1, A2, and A3) due to the different orientations of HO_2_^−^.

Firstly, the three intermediates IM1(A1), IM1(A2), and IM1(A3) can be formed barrierlessly, which are stabilized by about 28.57, 31.11 and 30.01 kcal/mol relative to the separated reactants, respectively. Meanwhile, the relevant transition states for their conversions have been located. As a result, the forward (reverse) free energy barriers are 3.56(5.69) and 6.45(5.70) kcal/mol for the conversions from IM1(A1) to IM1(A2) and from IM1(A2) to IM1(A3), respectively. Therefore, IM1(A2) is the most favorable intermediate, both thermodynamically and kinetically. Subsequently, the decomposition of the C-O bond connecting two six-membered rings occurs easily. Here, the calculated free energy barriers for the decomposition processes of the above three intermediates are 0.96, 0.29 and 0.29 kcal/mol. In aqueous solution, the corresponding free energy barrier is only 0.82 kcal/mol for IM1(A2). Obviously, low energy barriers suggest that the C-O bond can be readily cleaved, producing a new intermediate containing an O-O bond. As shown in Figure 5, taking the most stable IM1(A2) as an example, the second intermediate IM2(A2) can be formed after the cleavage of the C-O bond. Finally, the O-O bond of IM2(A2) can be homolytically cleaved via transition state TS2(A2), resulting in the formation of OH radical. Here, the calculated free energy barrier is 14.16 kcal/mol for the cleavage of the O-O bond.

Overall, the nucleophilic addition process is more favorable kinetically compared with the above nucleophilic substitution process.

### 2.3. Substitution Effects

To further explore the reactivity of the organic hydroperoxides with TCDD and its derivatives, the substitution effects including F-, Br-, and CH_3_- substitutes have been investigated.

**Figure 6:**
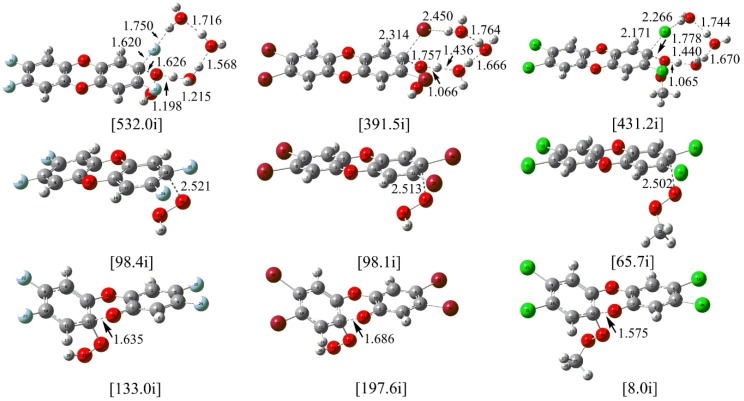
Calculated TSs in the nucleophilic substitution/addition of TCDD and its derivatives by hydroperoxides and their anions (top and middle/bottom), where F-, Br-, and CH_3_- substitutions are shown from left to right, respectively.

For the neutral H_2_O_2_, its reactions with the F- and Br-substituted TCDD have been investigated as well as the reaction between TCDD and the CH_3_-substituted H_2_O_2_. Here, the NAP involving three water molecules is considered since it is the rate-determining step as mentioned above. As shown in Figure 6, the located transition states are similar to the TS1(3w) mentioned above. For the reactions of F- and Br- substituted cases, the calculated free energy barriers are lower by 0.68 and 1.44 kcal/mol than those resulting before substitution. As for the reaction of the CH_3_-substituted H_2_O_2_ with TCDD, the free energy barrier is higher by 3.54 kcal/mol than that before substitution. Therefore, for the reaction involving neutral organic hydroperoxides, the energy barriers have not been significantly influenced upon substitution.

As for the above nucleophilic substitution reactions involving the anion of organic hydroperoxides, taking model S1 for example, the corresponding transition states have been given in Figure 6. For the reactions of HO_2_^−^ with F- and Br- substituted TCDD, the calculated free energy barriers are higher by 1.32 and 0.27 kcal/mol than the reaction before substitution. Similarly, for the reaction of the CH_3_^−^ substituted HO_2_^−^ with TCDD, the calculated free energy barrier is higher by about 3.13 kcal/mol than the reaction before substitution. Therefore, it is also feasible for the reaction to take place between the anions of the organic hydroperoxides and TCDD as well as its derivatives.

As for the nucleophilic addition reaction, the addition mode A2 has been explored. As shown in Figure 6, the corresponding transition states associated with the C-O bond cleavage have been located. Similar to the reaction before substitution, all the intermediates and transition states are lower in energy than the separated reactants. Moreover, the free energy barriers are only 0.29, 0.37, and 1.38 kcal/mol for the C-O bond cleavage process, respectively. Therefore, the feasibility of the nucleophilic addition reaction has been confirmed once again.

**Figure 7:**
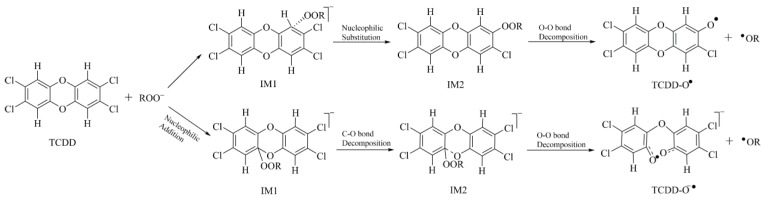
Proposed reaction mechanism for the reaction of ROO^−^(R=H or alkyl groups) with TCDD.

In summary, organic hydroperoxides, especially for their anions, can react with TCDD and its derivatives. Taking the reaction of ROO^−^(R=H or alkyl groups) with TCDD for example, the reaction mechanisms between them can be proposed below. As displayed in Figure 7, on the one hand, the reaction can occur through nucleophilic substitution mechanism to produce an intermediate containing an O-O bond. On the other hand, nucleophilic addition mechanism is also feasible for the reaction, leading to the decomposition of the C-O bond of TCDD and the formation of an intermediate containing an O-O bond. Finally, the O-O bond in these intermediates can be cleaved homolytically to produce different radicals depending on the introduced substitutes. Given the fact that these highly reactive radicals can cause oxidative damages to organisms, the above reaction mechanisms can be used to partially explain the potential toxicity mechanism of TCDD-like environmental contaminants. Certainly, more complicated experiments are required to further verify the above findings.

## 3. Computational Details

All the geometries have been fully optimized at the B3LYP/6-311++G(d,p) level of theory, where the reliability of the method has been verified by numerous systems [39,40,41,42,43,44,45,46,47,48,49,50]. Subsequently, vibrational frequency analysis has been carried out to identify the nature of the optimized species. For the located transition states (TSs), intrinsic reaction coordinate (IRC) [51,52] calculations have also been performed to further verify their correctness. For comparison, M06-2X/6-311++G(d,p) level of theory has also been employed for the direct nucleophilic substitution process involving neutral H_2_O_2_. As mentioned below, the calculated results are well consistent with the results of the B3LYP level. Thus, considering the compromise between computational accuracy and cost, the results at the B3LYP/6-311++G(d,p) level of theory have been mainly discussed if not otherwise noted.

To evaluate bulk solvent effects on the nucleophilic attack process (NAP), full optimizations have been performed for the selected species in aqueous solution employing the solvent model density (SMD) [53] solvation model. To explore the positive role of water molecules in the reaction, one, two, and three explicit water molecules have been introduced. Here, similar to previous studies on water-assisted proton transfer systems [33,34,54], the introduced water molecules have been located between the proton donor and proton acceptor through intermolecular H-bonds. Note that more water molecules without participating in the proton transfer have a slight influence on the reduction of the barrier heights [54].

To characterize the formation and nature of the intermolecular H-bonds formed in the intermediates, atoms in molecules (AIM) theory was employed based on the optimized geometries. In the AIM analyses [55], the locations of the bond critical point (BCP) and ring critical point (RCP) denote the presence of the interatomic interactions and the formation of a ring structure, respectively. Moreover, the nature of the H-bonding interaction can be predicted from the topological parameters of the electron density (*ρ*_bcp_) at the BCP of the H-bonds, e.g., the Laplacian of electron density (∇^2^*ρ*_bcp_) and energy density (*H*_bcp_) including the kinetic and potential energy density (*G*_bcp_ and *V*_bcp_).

All calculations were performed by using the Gaussian 09 program [56].

## 4. Conclusions

In this study, the reactivity of H_2_O_2_ and its anion toward TCDD and its selected derivatives has been systematically investigated theoretically. It was found that the neutral H_2_O_2_ is relatively difficult to react with TCDD even though the reaction process is assisted by explicit water molecules. On the contrary, the anion of H_2_O_2_ can easily react with TCDD, exhibiting the pH dependence of the title reaction. Overall, HO_2_^−^ can react with TCDD via two different reaction mechanisms, i.e., nucleophilic substitution and nucleophilic addition. For the former, the terminal O atom of HO_2_^−^ nucleophilically attacks the C atom of the C-Cl bond in TCDD to form an intermediate containing an O-O bond, accompanying the dissociation of the chlorine atom. For the latter, the terminal O atom of HO_2_^−^ can be easily attached to the C atom of the C-O bond in TCDD, resulting in the decomposition of the C-O bond and the formation of a new intermediate containing an O-O bond. Finally, different radicals can be produced via the cleavage of the O-O bond in those formed intermediates. Given the fact that the produced highly reactive radicals can lead to potential damage to organisms, the reaction mechanisms herein are expected to provide an alternative elucidation for the potential toxicity mechanism of TCDD-like environmental contaminants. Certainly, related experiments are highly desirable to further confirm the present findings.

## Figures and Tables

**Table 1:** Free energy barriers for the direct nucleophilic substitution of TCDD by H_2_O_2_ in different attack modes *^a^*.

Attack Modes	ΔG*_1_	ΔG*_2_	ΔG*_2_ − ΔG*_1_
Mode 1	62.43/62.22	63.63	1.20
Mode 2	62.39/62.77	61.47	−0.91
Mode 3	64.04/64.33	62.97	−1.07
Mode 4	61.63/62.89	61.27	−0.36

*^a^* All units are in kcal/mol. ΔG*_1_ and ΔG*_2_ refer to the results in the gas phase and in aqueous solution, respectively. The data behind the slash are the results at the M06-2X/6-311++G(d,p) level of theory.

**Table 2:** Calculated relative energy (Δ*E*), enthalpy changes (Δ*H*), and Gibbs free energy changes (Δ*G*) for the available intermediates, transition states, and products relative to the isolated reactants in the different reaction pathways involving neutral H_2_O_2_ *^a^*.

Pathways	Parameters	IM1	TS1	IM2	TS2	Pro
A	Δ*E*	−1.52	52.10	−7.82	−2.93(4.89)	−2.43
	Δ*H*	−0.83	51.47	−7.61	−3.37	−1.48
	ΔG	4.26	62.43	−0.33	5.94	−3.75
B	Δ*E*	−6.83	41.75	−13.74	−7.92(5.82)	−9.46
	Δ*H*	−6.55	40.64	−14.06	−9.14	−9.03
	ΔG	7.93	60.94	1.82	10.35	−2.58
C	Δ*E*	−13.82	31.84	−20.31	−9.04(11.27)	−15.90
	Δ*H*	−14.16	29.92	−21.07	−10.49	−15.94
	ΔG	10.27	60.08	2.70	16.79	−1.16
D	Δ*E*	−21.31	23.72	−25.79	−12.01(13.78)	−21.46
	Δ*H*	−22.00	21.37	−27.13	−13.89	−22.83
	ΔG	10.26	59.91	5.88	22.25	3.11

*^a^* All the units are in kcal/mol. The data in parentheses are the results relative to the corresponding IM2(nw).

**Table 3:** Calculated BDEs of the O-O bond in H_2_O_2_ and the second intermediates *^a^*.

Species	H_2_O_2_	IM2(0w)	IM2(1w)	IM2(2w)	IM2(3w)
BDE	48.62/44.08	20.32/6.14	19.81/5.03	18.89/5.14	18.96/4.96

*^a^* All the units are in kcal/mol. The data behind the slash refer to the adiabatic BDEs.

## Supplementary Materials

The following are available online. Figure S1: Molecular graphs of the intermediates (IM) and transition states (TS) in the different reaction pathways involving neutral H_2_O_2_. Table S1: Topological parameters for the intermediates in the different reaction pathways involving neutral H_2_O_2_. Table S2: Cartesian coordinates of the optimized species in the present study. Table S3: Calculated energy parameters for the optimized species in the present study.

## References

[1] Zou L.L., Ni Y.W., Gao Y., Tang F.M., Jin J., Chen J.P. (2018). Spatial variation of PCDD/F and PCB emissions and their composition profiles in stack flue gas from the typical cement plants in China. Chemosphere.

[2] Sappington E.N., Balasubramani A., Rifai H.S. (2015). Polychlorinated dibenzo-*p*-dioxins and polychlorinated dibenzofurans (PCDD/Fs) in municipal and industrial effluents. Chemosphere.

[3] Wang X.L., Ni Y.W., Zhang H.J., Zhang X.P., Chen J.P. (2012). Formation and emission of PCDD/Fs in Chinese non-wood pulp and paper mills. Environ. Sci. Technol..

[4] Wang X., Zhang H., Ni Y., Du Q., Zhang X., Chen J. (2014). Kinetics of PCDD/Fs formation from non-wood pulp bleaching with chlorine. Environ. Sci. Technol..

[5] Salamanca M., Chandia C., Hernandez A. (2016). Impact of forest fires on the concentrations of polychlorinated dibenzo-*p*-dioxin and dibenzofurans in coastal waters of central Chile. Sci. Total Environ..

[6] Dwyer H., Themelis N.J. (2015). Inventory of US 2012 dioxin emissions to atmosphere. Waste Manag..

[7] Heeb N.V., Rey M.D., Zennegg M., Haag R., Wichser A., Schmid P., Seiler C., Honegger P., Zeyer K., Mohn J. (2015). Biofuel-promoted polychlorinated dibenzodioxin/furan formation in an iron-catalyzed diesel particle filter. Environ. Sci. Technol..

[8] Heeb N.V., Zennegg M., Haag R., Wichser A., Schmid P., Seiler C., Ulrich A., Honegger P., Zeyer K., Emmenegger L. (2013). PCDD/F formation in an iron/potassium-catalyzed diesel particle filter. Environ. Sci. Technol..

[9] Altarawneh M., Dlugogorski B.Z., Kennedy E.M., Mackie J.C. (2009). Mechanisms for formation, chlorination, dechlorination and destruction of polychlorinated dibenzo-*p*-dioxins and dibenzofurans (PCDD/Fs). Prog. Energy Combust. Sci..

[10] Hidayat A., Tachibana S. (2013). Degradation of 2,4,8-trichlorodibenzofuran by a new isolate of *Cerrena* sp. F0607. Int. Biodeter. Biodegr..

[11] Govindan M., Moon I.S. (2015). Expeditious removal of PCDD/Fs from industrial waste incinerator fly ash using electrogenerated homogeneous Ag (II) ions. Chem. Eng. J..

[12] Palanisami N., Chung S.J., Moon I.S. (2015). Cerium (IV)-mediated electrochemical oxidation process for removal of polychlorinated dibenzo-*p*-dioxins and dibenzofurans. J. Ind. Eng. Chem..

[13] Liljelind P., Unsworth J., Maaskant O., Marklund S. (2001). Removal of dioxins and related aromatic hydrocarbons from flue gas streams by adsorption and catalytic destruction. Chemosphere.

[14] Finocchio E., Busca G., Notaro M. (2006). A review of catalytic processes for the destruction of PCDD and PCDF from waste gases. Appl. Catal. B.

[15] Daikoku T., Takemoto M., Yoshida Y., Okuda T., Takahashi Y., Ota K., Tokuoka F., Kawaguchi A.T., Shiraki K. (2015). Decomposition of organic chemicals in the air and inactivation of aerosol-associated influenza infectivity by photocatalysis. Aerosol Air Qual. Res..

[16] Huang L.Y., Su G.J., Liu Y.X., Li L.W., Liu S., Lu H.J., Zheng M.H. (2014). Effect of NiFe_2_O_4_ on PCDF byproducts formation during thermal degradation of decachlorobiphenyl. RSC Adv..

[17] Yu M.F., Lin X.Q., Li X.D., Chen T., Yan J.H. (2016). Catalytic decomposition of PCDD/Fs over nano-TiO_2_ based V_2_O_5_/CeO_2_ catalyst at low temperature. Aerosol Air Qual. Res..

[18] Hung P.C., Chang S.H., Ou-Yang C.C., Chang M.B. (2016). Simultaneous removal of PCDD/Fs, pentachlorophenol and mercury from contaminated soil. Chemosphere.

[19] Hajizadeh Y., Onwudili J.A., Williams P.T. (2011). Removal potential of toxic 2,3,7,8-substituted PCDD/F from incinerator flue gases by waste-derived activated carbons. Waste Manag..

[20] Kawashima A., Katayama M., Matsumoto N., Honda K. (2011). Physicochemical characteristics of carbonaceous adsorbent for dioxin-like polychlorinated biphenyl adsorption. Chemosphere.

[21] Zhang K., Sun S.M., Zhang H. (2015). Mechanism and kinetic study on the ring-opening degradation of 2,3,7,8-tetrachlorinated dibenzofuran initiated by OH radicals in waste incineration. RSC Adv..

[22] Sun X.M., Zhang C.X., Zhao Y.Y., Bai J., Zhang Q.Z., Wang W.X. (2012). Atmospheric chemical reactions of 2,3,7,8-tetrachlorinated dibenzofuran initiated by an OH radical: Mechanism and kinetics study. Environ. Sci. Technol..

[23] Zhang C.X., Sun X.M., Xu Y.S., Qi C.S., Zhang J.H. (2014). Atmospheric chemistry of 2,3,7,8-TCDD/F: Mechanism and kinetics study. J. Environ. Chem. Eng..

[24] Pan W.X., Qi Y.Y., Wang R.X., Han Z., Zhang D.J., Zhan J.H. (2013). Adsorption of TCDD with 1-butyl-3-methylimidazolium dicyanamide ionic liquid: A combined molecular dynamics simulation and quantum chemistry study. Chemosphere.

[25] Zhou Q.X., Yong Y.L., Ju W.W., Su X.Y., Li X.H., Wang C.Y., Fu Z.B. (2018). DFT study of the adsorption of 2,3,7,8-tetrachlorodibenzofuran (TCDF) on vacancy-defected graphene doped with Mn and Fe. Curr. Appl. Phys..

[26] Weber R., Sakurai T., Hagenmaier H. (1999). Formation and destruction of PCDD/F during heat treat treatment of fly ash samples from fluidized bed incinerators. Chemosphere.

[27] Lundin L., Marklund S. (2005). Thermal degradation of PCDD/F in municipal solid waste ashes in sealed glass ampules. Environ. Sci. Technol..

[28] Zhu B.Z., Kalyanaraman B., Jiang G.B. (2007). Molecular mechanism for metal-independent production of hydroxyl radicals by hydrogen peroxide and halogenated quinines. Proc. Natl. Acad. Sci. USA.

[29] Zhu B.Z., Zhao H.T., Kalyanaraman B., Liu J., Shan G.Q., Du Y.G., Frei B. (2007). Mechanism of metal-independent decomposition of organic hydroperoxides and formation of alkoxyl radicals by halogenated quinones. Proc. Natl. Acad. Sci. USA.

[30] Zhu B.Z., Shan G.Q., Huang C.H., Kalyanaraman B., Mao L., Du Y.G. (2009). Metal-independent decomposition of hydroperoxides by halogenated quinones: Detection and identification of a quinone ketoxy radical. Proc. Natl. Acad. Sci. USA.

[31] Huang C.H., Ren F.R., Shan G.Q., Qin H., Mao L., Zhu B.Z. (2015). Molecular mechanism of metal-independent decomposition of organic hydroperoxides by halogenated quinoid carcinogens and the potential biological implications. Chem. Res. Toxicol..

[32] Yin R., Zhang D., Song Y., Zhu B.Z., Wang H. (2013). Potent DNA damage by polyhalogenated quinones and H_2_O_2_ via a metal-independent and intercalation-enhanced oxidation mechanism. Sci. Rep..

[33] Li P., Wang W.H., Sun Q., Li Z., Du A.J., Bi S.W., Zhao Y. (2013). Insights into the mechanism of the reaction between tetrachloro-*p*-benzoquinone and hydrogen peroxide and their implications in the catalytic role of water molecules in producing the hydroxyl radical. Chem. Phys. Chem..

[34] Li P., Guo C., Feng W.L., Sun Q., Wang W.H. (2017). A DFT study on the reaction mechanism between tetrachloro-*o*-benzoquinone and H_2_O_2_ and an alternative reaction approach to produce the hydroxyl radical. RSC Adv..

[35] Arulmozhiraja S., Morita M. (2004). Electron affinities and reductive dechlorination of toxic polychlorinated dibenzofurans: A density functional theory study. J. Phys. Chem. A.

[36] Zhao Y.Y., Tao F.M., Zeng E.Y. (2007). Structures, reductive dechlorination, and electron affinities of selected polychlorinated dibenzo-*p*-dioxins: Density functional theory study. J. Phys. Chem. A.

[37] Guo C., Wang W.H., Feng W.L., Li P. (2017). Insights into the one-electron reduction behavior of tetrachloro-*o*-benzoquinone: A DFT and molecular dynamics study. RSC Adv..

[38] Li P., Wang W.H., Sun H.T., Bi S.W. (2013). A DFT study on the electron affinity of tetrachloro-*p*-benzoquinone: Toward to understanding its electron-accepting ability in solution. Comput. Theor. Chem..

[39] Wei W.J., Wang W.H., Xu K.N., Feng W.L., Li X.P., Li P. (2018). Theoretical insights into the reaction mechanisms between 2,3,7,8-tetrachlorodibenzofuran and the methylidyne radical. RSC Adv..

[40] Zhao Y., Wang W.H., Feng W.L., Wang W.L., Li P. (2018). Theoretical insights into the interaction mechanisms between nitric acid and nitrous oxide initiated by an excess electron. J. Phys. Chem. A.

[41] Wang W.H., Feng W.L., Wang W.L., Li P. (2018). Theoretical investigations on the reactivity of methylidyne radical toward 2,3,7,8-tetrachlorodibenzo-*p*-dioxin: A DFT and molecular dynamics study. Molecules.

[42] Wang W.H., Feng W.L., Wang W.L., Li P. (2018). Theoretical insights into the electron capture behavior of H_2_SO_4_···N_2_O complex: A DFT and molecular dynamics study. Molecules.

[43] Xu F., Shi X., Zhang Q., Wang W. (2016). Mechanism for the growth of polycyclic aromatic hydrocarbons from the reactions of naphthalene with cyclopentadienyl and indenyl. Chemosphere.

[44] Zhuang S., Lv X., Pan L., Lu L., Ge Z., Wang J., Wang J., Liu J., Liu W., Zhang C. (2017). Benzotriazole UV 328 and UV-P showed distinct antiandrogenic activity upon human CYP3A4-mediated biotransformation. Environ. Pollut..

[45] Zhu L., Shi X., Sun Y., Zhang Q., Wang W. (2017). The growth mechanism of polycyclic aromatic hydrocarbons from the reactions of anthracene and phenanthrene with cyclopentadienyl and indenyl. Chemosphere.

[46] Xu K., Wang W., Wei W., Feng W., Sun Q., Li P. (2017). Insights into the reaction mechanism of Criegee intermediate CH_2_OO with methane and implications for the formation of methanol. J. Phys. Chem. A.

[47] Wang W.H., Zhang X.X., Li P., Sun Q., Li Z., Ren C., Guo C. (2015). CO_2_ capture and separation from N_2_/CH_4_ mixtures by Co@B_8_/Co@B_8_^−^ and M@B_9_/M@B_9_^−^ (M = Ir, Rh, Ru) clusters: A theoretical study. J. Phys. Chem. A.

[48] Feng W.L., Ren C., Wang W.H., Guo C., Sun Q., Li P. (2016). An identification of the C–C bonding spin adduct in the spin trapping of *N*-methyl benzohydroxamic acid radical by 5,5-dimethyl-1-pyrroline-*N*-oxide. Theor. Chem. Acc..

[49] Feng W.L., Ren C., Wang W.H., Guo C., Sun Q., Li P. (2016). Theoretical studies on the spin trapping of the 2-chloro-5-hydroxy-1,4-benzoquinone radical by 5,5-dimethyl-1-pyrroline *N*-oxide (DMPO): The identification of the C–O bonding spin adduct. RSC Adv..

[50] Wang W.H., Guo C., Feng W.L., Sun Q., Li P. (2017). Theoretical insights into the reaction mechanism between tetrachloro-*o*-benzoquinone and *N*-methyl benzohydroxamic acid. RSC Adv..

[51] Gonzalez C., Schlegel H.B. (1989). An improved algorithm for reaction path following. J. Chem. Phys..

[52] Gonzalez C., Schlegel H.B. (1990). Reaction path following in mass-weighted internal coordinates. J. Phys. Chem..

[53] Marenich A.V., Cramer C.J., Truhlar D.G. (2009). Universal solvation model based on solute electron density and on a continuum model of the solvent defined by the bulk dielectric constant and atomic surface tensions. J. Phys. Chem..

[54] Li P., Bu Y.X. (2004). Multiwater-assisted proton transfer study in glycinamide using density functional theory. J. Phys. Chem. B.

[55] Bader R.F.W. (1985). Atoms in molecules. Acc. Chem. Res..

[56] Frisch M.J., Trucks G.W., Schlegel H.B., Scuseria G.E., Robb M.A., Cheeseman J.R., Scalmani G., Barone V., Mennucci B., Petersson G.A. (2009). Gaussian 09.

