# Supplementary material for: Theoretical Investigations on the Reactivity of Hydrogen Peroxide toward 2,3,7,8-Tetrachlorodibenzo-p-dioxin
Source: Molecules. 2018 Oct 31;23(11):2826. doi: 10.3390/molecules23112826 (PMC6278397; doi:10.3390/molecules23112826)
Supplement: Supplementary file 1 [file molecules-23-02826-s001.zip › molecules-380137-SI.pdf]

Molecules 2018, 23, 2826; doi:10.3390/molecules23112826 www.mdpi.com/journal/molecules

Theoretical Investigations on the Reactivity of Hydrogen Peroxide toward 2,3,7,8-Tetrachlorodibenzo-*p*-dioxin

Weihua Wang *, Yuhua Wang, Wenling Feng, Wenliang Wang and Ping Li *

Key Laboratory of Life-Organic Analysis, School of Chemistry and Chemical Engineering, Qufu Normal University, Qufu 273165, China; wyhqfnu@163.com (Y.W.); wlfengqf@163.com (W.F.); wlwangqf@126.com (W.W.)

* Correspondence: wwh78@163.com (W.W.); lignip@163.com

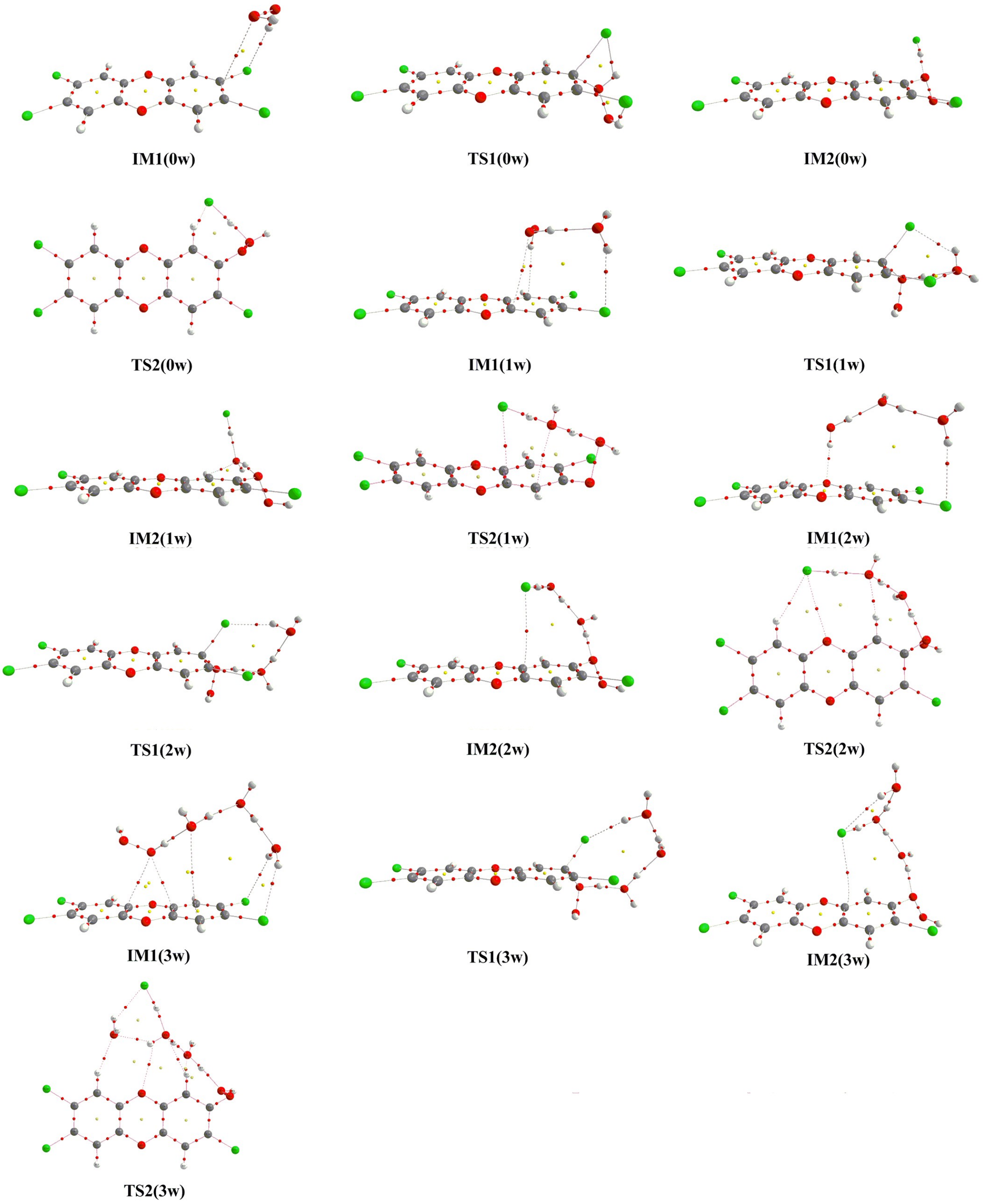

(P.L.)

Figure S1. Molecular graphs of the intermediates (IM) and transition states (TS) in the different reaction pathways involving neutral H_2O_2. BCP and RCP are represented by red and yellow dots, respectively.

Table S1. Topological parameters for the intermediates in the different reaction pathways involving neutral H_2O_2[a]

Species	BCP	ρ_{bcp}	$\nabla^2\rho_{bcp}$	V_{bcp}	G_{bcp}	H_{bcp}
IM1(0w)	Cl17···H23	0.0103	0.0332	-0.0047	0.0065	0.0018
	C11···O25	0.0029	0.0099	-0.0016	0.0020	0.0005
	O24···O25	0.2735	0.0374	-0.3636	0.1865	-0.1771
IM2(0w)	O24···O25	0.2511	0.1360	-0.3292	0.1816	-0.1476
	O25···H26	0.0244	0.0839	-0.0165	0.0188	0.0022
IM1(1w)	C9···H23	0.0058	0.0156	-0.0026	0.0033	0.0006
	O24···O25	0.2723	0.0416	-0.3616	0.1860	-0.1756
	Cl18···H29	0.0087	0.0294	-0.0039	0.0056	0.0017
	H26···O27	0.0313	0.1094	-0.0254	0.0264	0.0010
IM2(1w)	O25···H26	0.0248	0.0930	-0.0190	0.0211	0.0021
	O27···H29	0.0362	0.1135	-0.0280	0.0282	0.0002
	H21···O27	0.0059	0.0209	-0.0035	0.0044	0.0009
	O24 - O25	0.2480	0.1454	-0.3240	0.1802	-0.1438
IM1(2w)	O13···H23	0.0148	0.0522	-0.0101	0.0116	0.0015
	H26···O27	0.0353	0.1228	-0.0305	0.0306	0.0001
	Cl18···H32	0.0092	0.0305	-0.0041	0.0058	0.0018
	H29···O30	0.0292	0.1086	-0.0237	0.0254	0.0017
	O24···O25	0.2710	0.0443	-0.3591	0.1851	-0.1740
IM2(2w)	O24 - O25	0.2492	0.1433	-0.3261	0.1810	-0.1452
	O25···H26	0.0264	0.1009	-0.0208	0.0230	0.0022
	O30···H32	0.0454	0.1260	-0.0381	0.0348	-0.0033
	O27···H29	0.0346	0.1206	-0.0297	0.0299	0.0002
IM1(3w)	O24···O25	0.2719	0.0438	-0.3613	0.1861	-0.1752
	H26···O27	0.0396	0.1309	-0.0355	0.0341	-0.0014
	H29···O30	0.0347	0.1233	-0.0301	0.0305	0.0003
	Cl17···H33	0.0064	0.0209	-0.0031	0.0042	0.0011
	H32···O34	0.0339	0.1188	-0.0288	0.0292	0.0005
	Cl18···H35	0.0098	0.0319	-0.0046	0.0063	0.0017
IM2(3w)	O25···H26	0.0265	0.1000	-0.0209	0.0229	0.0021
	O30···H31	0.0817	0.1243	-0.0813	0.0562	-0.0251
	O27···H29	0.0337	0.1220	-0.0292	0.0299	0.0006
	H32···O34	0.0264	0.0968	-0.0205	0.0224	0.0018
	Cl18···H35	0.0104	0.0342	-0.0052	0.0069	0.0017
	O24 - O25	0.2483	0.1458	-0.3245	0.1805	-0.1440

[a] Atomic numbering refers to Figure 2. The ρ_{bcp}, $\nabla^2\rho_{bcp}$, V_{bcp}, G_{bcp}, and H_{bcp} is electron density, the Laplacian of the electron density, potential energy density, kinetic energy density, and energy density at the BCP, respectively.

Table S2. Cartesian coordinates of the optimized species in the present study*

Species	x	y	z	Species	x	y	z
TCDD				H_2O_2			
C	0.00000000	3.57881500	0.69850100	O	0.00000000	0.72735500	-0.05197800
C	0.00000000	2.36878800	1.39107000	H	0.82815300	0.90273800	0.41582100
C	0.00000000	1.16979600	0.69781000	O	0.00000000	-0.72735500	-0.05197800
C	0.00000000	1.16979600	-0.69781000	H	-0.82815300	-0.90273800	0.41582100
C	0.00000000	2.36878800	-1.39107000				
C	0.00000000	3.57881500	-0.69850100				
C	0.00000000	-1.16979600	-0.69781000				
C	0.00000000	-1.16979600	0.69781000				
C	0.00000000	-2.36878800	-1.39107000				
C	0.00000000	-2.36878800	1.39107000				
C	0.00000000	-3.57881500	-0.69850100				
C	0.00000000	-3.57881500	0.69850100				
O	0.00000000	0.00000000	-1.42570200				
O	0.00000000	0.00000000	1.42570200				
Cl	0.00000000	5.06339400	-1.61505000				
Cl	0.00000000	5.06339400	1.61505000				
Cl	0.00000000	-5.06339400	-1.61505000				
Cl	0.00000000	-5.06339400	1.61505000				
H	0.00000000	2.35436000	-2.47284400				
H	0.00000000	2.35436000	2.47284400				
H	0.00000000	-2.35436000	2.47284400				
H	0.00000000	-2.35436000	-2.47284400				
TS1				TS2			
C	-4.05553500	-0.73295300	0.06731600	C	-4.04797400	-0.73815000	0.04008500
C	-2.81973100	-1.37546100	0.13175900	C	-2.81087500	-1.37880100	0.09772600
C	-1.64909000	-0.63789200	0.07880300	C	-1.64150600	-0.63734600	0.07639400
C	-1.70057300	0.75155100	-0.04226600	C	-1.69566100	0.75524900	-0.00799800
C	-2.92597000	1.39457700	-0.10794400	C	-2.92288400	1.39593800	-0.06759900
C	-4.10822000	0.65774800	-0.05292400	C	-4.10355600	0.65500100	-0.04244300
C	0.63806300	0.83522000	-0.05270400	C	0.64273400	0.84425500	-0.01533700
C	0.68947000	-0.55766300	0.06525900	C	0.69619000	-0.54841300	0.07006700
C	1.81117700	1.56630000	-0.12004400	C	1.81450000	1.57981300	-0.08135500
C	1.90709200	-1.21894700	0.10340800	C	1.91847700	-1.20474100	0.08471000
C	3.04269800	0.91390300	-0.07199800	C	3.05472500	0.94095400	-0.05775900
C	3.06607600	-0.47092400	-0.02596400	C	3.07003400	-0.44576700	-0.03491500
O	-0.56081500	1.51986400	-0.09632600	O	-0.55895800	1.52806000	-0.03029500
O	-0.45165800	-1.31899100	0.15053900	O	-0.44346400	-1.31691000	0.14439600
Cl	-5.62682000	1.51313200	-0.13717500	Cl	-5.62402100	1.50861200	-0.11631000
Cl	-5.50380300	-1.70301000	0.13951500	Cl	-5.49421900	-1.71409300	0.07300000
Cl	4.53007300	1.84239700	-0.12479400	Cl	4.52085500	1.87573200	-0.13462600
Cl	4.48510500	-1.42124600	-1.54240200	Cl	4.51341600	-1.35855800	-1.58281500
H	-2.95372700	2.47191500	-0.20157500	H	-2.95283100	2.47531200	-0.13291100
H	-2.76353500	-2.45188700	0.22411300	H	-2.75270100	-2.45728100	0.16009500
H	1.93924400	-2.29648300	0.18234800	H	1.95599100	-2.28435500	0.12560400
H	1.76580100	2.64433400	-0.20499500	H	1.76315600	2.65893900	-0.14821500
H	4.91605400	0.51491900	1.83709300	H	4.29814700	-1.05338500	2.85178200
O	4.74624900	-0.37769200	2.19822000	O	4.66922300	-0.43255700	2.20456500
O	4.34081300	-1.11606100	1.02957100	O	4.35090300	-1.13671000	0.97575100
H	5.08927900	-1.22413400	0.37332500	H	5.12886200	-1.08102100	0.35161500
TS3				TS4			
C	-3.98623300	-0.74402900	-0.04180000	C	-4.00173900	-0.75408300	-0.04494200
C	-2.73894300	-1.36684800	-0.01146400	C	-2.75131700	-1.37105700	-0.03494700

C	-1.58406100	-0.60575400	0.04389600	C	-1.59834700	-0.60590800	0.01831500
C	-1.66040400	0.78758300	0.06563700	C	-1.68263600	0.78691600	0.05983900
C	-2.89734700	1.41090800	0.03262000	C	-2.92287800	1.40449300	0.04682700
C	-4.06484400	0.65057000	-0.01869100	C	-4.08724100	0.63941600	-0.00376000
C	0.67502000	0.91329100	0.08277600	C	0.65349000	0.92669300	0.07612800
C	0.75053000	-0.47863600	0.07601400	C	0.73672500	-0.46692100	0.03755400
C	1.83769700	1.66855800	0.05529400	C	1.80827800	1.69138700	0.08533400
C	1.98701700	-1.11669300	0.03870900	C	1.97316100	-1.09641100	-0.00258300
C	3.08803000	1.05390500	0.02635800	C	3.06292200	1.07947200	0.05311300
C	3.13558100	-0.33500200	-0.04365300	C	3.10107900	-0.30080300	-0.04936000
O	-0.53609100	1.57761100	0.12317100	O	-0.56252500	1.58258000	0.11704100
O	-0.37255200	-1.26902500	0.08488700	O	-0.38599300	-1.26277400	0.03531700
Cl	-5.59815900	1.48170600	-0.05454100	Cl	-5.62533500	1.46406300	-0.01505200
Cl	-5.41478700	-1.74267000	-0.10758200	Cl	-5.42659200	-1.75933500	-0.10872800
Cl	4.53464600	2.01560700	0.02477700	Cl	4.50923700	2.04492400	0.09373200
Cl	4.51000500	-1.18214100	-1.65793100	Cl	4.56383500	-1.08428200	-1.67390500
H	-2.94547000	2.49147300	0.04883400	H	-2.97588000	2.48452000	0.07731400
H	-2.66268700	-2.44587100	-0.02985200	H	-2.66987900	-2.44935700	-0.06752300
H	2.02317800	-2.19319500	-0.07249800	H	2.02983700	-2.17479700	-0.04930800
H	1.76857700	2.74878400	0.07262300	H	1.73341700	2.77057100	0.12414200
H	3.03477100	-1.64789500	2.01613100	H	4.25450800	-1.38968600	2.75855200
O	4.00333800	-1.60429600	2.15573100	O	4.06213900	-1.91379500	1.96518300
O	4.42926600	-0.96269800	0.93014600	O	4.46635800	-0.98605100	0.94364200
H	4.79869200	-1.64570600	0.29879700	H	4.87972700	-1.51138000	0.18545000
	IM1(0w)				TS1(0w)		
C	4.13791100	0.63936800	0.20817000	C	-4.05553500	-0.73295300	0.06731600
C	2.95035400	1.36954800	0.17588300	C	-2.81973100	-1.37546100	0.13175900
C	1.74393000	0.72558800	-0.04082900	C	-1.64909000	-0.63789200	0.07880300
C	1.71304600	-0.65684100	-0.22856100	C	-1.70057300	0.75155100	-0.04226600
C	2.88902300	-1.38727700	-0.19790700	C	-2.92597000	1.39457700	-0.10794400
C	4.10710400	-0.74449500	0.02064300	C	-4.10822000	0.65774800	-0.05292400
C	-0.61098700	-0.57068900	-0.48159300	C	0.63806300	0.83522000	-0.05270400
C	-0.58051600	0.81228700	-0.29338800	C	0.68947000	-0.55766300	0.06525900
C	-1.81625500	-1.21212600	-0.71381000	C	1.81117700	1.56630000	-0.12004400
C	-1.75483900	1.54548100	-0.33677900	C	1.90709200	-1.21894700	0.10340800
C	-2.99804500	-0.47664800	-0.75771700	C	3.04269800	0.91390300	-0.07199800
C	-2.97119400	0.90498500	-0.56631400	C	3.06607600	-0.47092400	-0.02596400
O	0.53330100	-1.33524300	-0.44637000	O	-0.56081500	1.51986400	-0.09632600
O	0.59599400	1.48862000	-0.06251200	O	-0.45165800	-1.31899100	0.15053900
Cl	5.56223800	-1.70605300	0.05118500	Cl	-5.62682000	1.51313200	-0.13717500
Cl	5.63338800	1.49399600	0.48517000	Cl	-5.50380300	-1.70301000	0.13951500
Cl	-4.49674100	-1.33144300	-1.05692100	Cl	4.53007300	1.84239700	-0.12479400
Cl	-4.42685600	1.86685100	-0.60738100	Cl	4.48510500	-1.42124600	-1.54240200
H	2.85097100	-2.45851700	-0.34401500	H	-2.95372700	2.47191500	-0.20157500
H	2.95999500	2.44162400	0.32002100	H	-2.76353500	-2.45188700	0.22411300
H	-1.71576000	2.61638400	-0.18862000	H	1.93924400	-2.29648300	0.18234800
H	-1.82714400	-2.28440400	-0.85595400	H	1.76580100	2.64433400	-0.20499500
H	-5.42250100	-1.31222700	1.34389900	H	4.91605400	0.51491900	1.83709300
O	-5.57816100	-1.18552000	2.29290500	O	4.74624900	-0.37769200	2.19822000
O	-4.47335000	-0.30571300	2.63150900	O	4.34081300	-1.11606100	1.02957100
H	-4.94952900	0.50792900	2.84869900	H	5.08927900	-1.22413400	0.37332500
	IM2(0w)				TS2(0w)		
C	3.99569400	0.90666300	0.11483800	C	3.88757100	0.73753400	-0.18913500
C	2.69439800	1.39308800	0.23787800	C	2.62436200	1.24725700	-0.48797600
C	1.61720600	0.52911800	0.13602100	C	1.50489500	0.44267700	-0.35471600
C	1.83195900	-0.83005200	-0.09160700	C	1.64914700	-0.87262600	0.07872800
C	3.12062000	-1.31996400	-0.21471500	C	2.89584200	-1.39001200	0.37986900

C	4.20986200	-0.45501100	-0.11240900	C	4.02607900	-0.58561400	0.24623600
C	-0.47925200	-1.19970300	-0.07709200	C	-0.66657700	-1.18409700	-0.05676600
C	-0.69421700	0.16288400	0.14856700	C	-0.81630200	0.15044800	-0.50020400
C	-1.55299000	-2.07387900	-0.18884300	C	-1.77075400	-2.02530600	0.10933600
C	-1.98615600	0.64718200	0.26841900	C	-2.06577700	0.63715600	-0.76926900
C	-2.84862700	-1.58524200	-0.07770300	C	-3.03463900	-1.54418000	-0.15807000
C	-3.06847000	-0.22242000	0.14525600	C	-3.21717500	-0.20046000	-0.64267200
O	0.78468900	-1.72320700	-0.19926900	O	0.54673100	-1.70353700	0.22270900
O	0.34921800	1.05302800	0.26462300	O	0.27557500	0.97123200	-0.66042200
Cl	5.81570500	-1.11610400	-0.27416800	Cl	5.58280400	-1.26554900	0.63117800
Cl	5.32090700	2.03191000	0.25101500	Cl	5.26702300	1.78338900	-0.36997600
Cl	-4.20162900	-2.68057400	-0.22568700	Cl	-4.41076400	-2.55454600	0.06795800
Cl	-5.05167000	3.32154700	-0.76277700	Cl	-3.59611400	3.29951700	0.89890800
H	3.27193800	-2.37658300	-0.39061800	H	2.98304100	-2.41436900	0.71644700
H	2.51446300	2.44527200	0.41362100	H	2.50621000	2.26952100	-0.82161700
H	-2.15558800	1.70150200	0.44641500	H	-2.20885900	1.66500800	-1.07628300
H	-1.36392000	-3.12409400	-0.36661300	H	-1.60849600	-3.03743700	0.45480300
H	-5.53706500	-0.62785000	1.35624700	H	-5.98447500	1.25151200	0.09536800
O	-4.90954100	0.08918000	1.54189800	O	-5.15762600	1.01921400	0.54461900
O	-4.34263900	0.29270800	0.18753200	O	-4.37368300	0.23774700	-0.96724600
H	-4.84011500	2.10686100	-0.35148600	H	-4.44556000	2.12408000	0.70468100
	IM1(1w)				TS1(1w)		
C	-4.05964600	-0.72613200	-0.18366300	C	-4.34987100	-0.76511900	0.03673600
C	-2.84698300	-1.34977000	-0.47585100	C	-3.10238800	-1.38694500	0.06533500
C	-1.67547400	-0.61272100	-0.49944200	C	-1.94463800	-0.62762700	0.02918100
C	-1.70508900	0.75526800	-0.22960000	C	-2.02138300	0.76496700	-0.03880400
C	-2.90457700	1.38011000	0.06402200	C	-3.25963100	1.38706600	-0.06785800
C	-4.08866800	0.64377200	0.08749000	C	-4.42797200	0.62778900	-0.03009400
C	0.61645700	0.85803400	-0.49423700	C	0.31524900	0.88804400	-0.05701500
C	0.64514100	-0.51194400	-0.76497900	C	0.39331100	-0.50824200	0.00601900
C	1.79502800	1.58696900	-0.47100800	C	1.47722900	1.63272000	-0.09675300
C	1.85241100	-1.14461600	-1.00489400	C	1.61718300	-1.14912000	0.02454000
C	3.01589600	0.95327500	-0.70928300	C	2.72279400	0.99877000	-0.06831200
C	3.03990100	-0.41683100	-0.97024000	C	2.79049700	-0.39707700	-0.07995600
O	-0.55949000	1.52471000	-0.25009500	O	-0.89803000	1.55435800	-0.07654000
O	-0.50085300	-1.26867100	-0.80090200	O	-0.73668700	-1.29083900	0.06156700
Cl	-5.57726900	1.47331600	0.46029100	Cl	-5.96270400	1.45899300	-0.06756100
Cl	-5.51071200	-1.69401200	-0.16826100	Cl	-5.78059900	-1.76349300	0.08504500
Cl	4.47541300	1.90867400	-0.66670300	Cl	4.17793200	1.98321300	-0.15198000
Cl	4.54629100	-1.27002900	-1.24187900	Cl	4.08206000	-1.23094600	-1.64179000
H	-2.91253200	2.44111500	0.27458800	H	-3.30744600	2.46647800	-0.12049100
H	-2.81009400	-2.41054700	-0.68478900	H	-3.02618800	-2.46494400	0.11584000
H	1.86177400	-2.20826000	-1.20184000	H	1.66190100	-2.22834800	0.06775800
H	1.75525600	2.65189200	-0.28136300	H	1.41869800	2.71201000	-0.15352500
H	2.23607300	1.19872100	2.21342600	H	3.43384500	0.38087000	2.17266400
O	2.42271300	0.67343100	3.00425600	O	3.77437100	-0.51889600	2.34028200
O	1.86632900	-0.61271900	2.61750500	O	3.95233200	-1.02693500	0.99876800
H	2.68731600	-1.13501800	2.50411900	H	4.94177200	-0.81368200	0.75302500
O	4.33510000	-1.91458200	2.22922900	O	6.38673900	-0.68977300	0.33254900
H	4.99442200	-1.54555200	2.82659600	H	6.76872200	0.19389100	0.38178000
H	4.69773300	-1.80732800	1.34080100	H	6.23290100	-0.87296200	-0.61129900
	IM2(1w)				TS2(1w)		
C	4.07649400	0.98767200	0.26456400	C	3.82690500	0.51626300	-0.47808400
C	2.73864000	1.30066200	0.50382900	C	2.62391100	1.00568500	-0.98483600
C	1.75344500	0.35346700	0.28208100	C	1.45213000	0.29755400	-0.78072300
C	2.09754300	-0.91515200	-0.18390500	C	1.48467500	-0.89743500	-0.06873200
C	3.42298200	-1.23303400	-0.42405300	C	2.66936100	-1.39231300	0.44430500

C	4.42027500	-0.28392000	-0.20085300	C	3.85214800	-0.68563000	0.24023800
C	-0.16150200	-1.52745800	-0.18935500	C	-0.83278200	-1.10335200	-0.27053800
C	-0.50592200	-0.25544200	0.27731700	C	-0.87328500	0.12145100	-0.99427100
C	-1.14458600	-2.47675500	-0.43990200	C	-1.99790100	-1.81618300	0.02805700
C	-1.83513800	0.06219600	0.50185700	C	-2.07438700	0.64742900	-1.35376700
C	-2.47812800	-2.15408700	-0.22513300	C	-3.21732100	-1.28918800	-0.32647700
C	-2.82653300	-0.88289500	0.24167400	C	-3.30357700	-0.02230100	-1.03076500
O	1.14373000	-1.88622300	-0.41825800	O	0.32649600	-1.62965000	0.14004600
O	0.44523500	0.70481600	0.53424500	O	0.28422800	0.78573700	-1.30773800
Cl	6.07716600	-0.72758000	-0.51677900	Cl	5.33253600	-1.33146200	0.89265700
Cl	5.28270100	2.21207100	0.55851300	Cl	5.27845800	1.43513600	-0.76032400
Cl	-3.71864300	-3.34226800	-0.54904200	Cl	-4.66920500	-2.10677700	0.08901900
Cl	-4.54397100	4.37566800	-1.32946200	Cl	-0.93508000	1.22578900	2.18987500
H	3.67480000	-2.22118800	-0.78531200	H	2.66644100	-2.31893200	1.00220700
H	2.45852400	2.28199000	0.86279300	H	2.59432300	1.93689600	-1.53412500
H	-2.10594300	1.04637200	0.86444000	H	-2.13138600	1.60521600	-1.85367200
H	-0.85647600	-3.45336800	-0.80520800	H	-1.91338300	-2.74431000	0.57671600
H	-5.18059700	-1.80687600	1.35172100	H	-5.61413800	2.09589700	-0.23559900
O	-4.66614400	-1.05063700	1.67748400	O	-4.96584600	1.59754600	0.27858600
O	-4.14504600	-0.51762700	0.39087900	O	-4.41751100	0.50367500	-1.30189800
H	-4.55688100	1.33984500	0.73674200	H	-3.13305500	3.49356900	1.64829300
O	-4.50134200	2.28197700	0.96652300	O	-2.90543800	2.80174400	1.01690700
H	-5.10239000	2.41469900	1.70653200	H	-3.79375800	2.32382600	0.69910000
H	-4.54122900	3.48438500	-0.36235800	H	-2.02381200	2.05786300	1.55254100
	IM1(2w)				TS1(2w)		
C	4.03332400	-0.70940900	-0.52483600	C	-4.70086400	-0.76531500	-0.02644800
C	2.81187600	-1.19101900	-0.99442200	C	-3.45398900	-1.38698200	0.02298000
C	1.63168700	-0.79754800	-0.38802900	C	-2.29570200	-0.62731000	0.02202400
C	1.66398100	0.07658000	0.69651900	C	-2.37174200	0.76636300	-0.03116300
C	2.87146300	0.55245100	1.17401400	C	-3.60977800	1.38804000	-0.08076200
C	4.06417800	0.16445300	0.56395400	C	-4.77823000	0.62813700	-0.07828500
C	-0.66001000	-0.12205600	0.91873300	C	-0.03524200	0.88943500	-0.00135900
C	-0.68484500	-0.99020000	-0.17415200	C	0.04244000	-0.50696100	0.04788700
C	-1.82051200	0.15378100	1.61964800	C	1.12753600	1.63328300	-0.01317600
C	-1.88122900	-1.56312700	-0.57138100	C	1.26547400	-1.14823700	0.08041000
C	-3.03046000	-0.41270400	1.21916100	C	2.37372000	0.99967200	0.02927400
C	-3.05587700	-1.26699500	0.11723200	C	2.44443700	-0.39755600	0.01192300
O	0.50176300	0.50546900	1.31600600	O	-1.24929200	1.55646200	-0.03389500
O	0.44712000	-1.29318600	-0.88713800	O	-1.08920200	-1.29071200	0.07420400
Cl	5.56445200	0.79137800	1.19534300	Cl	-6.31246600	1.45952200	-0.14059400
Cl	5.49466600	-1.22794500	-1.32446600	Cl	-6.13201700	-1.76490500	-0.02238600
Cl	-4.47572500	-0.02075000	2.11522900	Cl	3.82990000	1.97904600	-0.03213800
Cl	-4.55557400	-1.97455000	-0.45593500	Cl	3.76038700	-1.22777200	-1.47685600
H	2.88232500	1.23113200	2.01639200	H	-3.65695900	2.46799700	-0.12156400
H	2.77477200	-1.86716600	-1.83792600	H	-3.37842600	-2.46548700	0.06247200
H	-1.89271300	-2.22583800	-1.42621300	H	1.30816400	-2.22791400	0.11383600
H	-1.78359800	0.82493800	2.46732800	H	1.07101600	2.71316900	-0.06057900
H	0.16725500	2.55778200	0.62853700	H	2.84782700	0.31857700	2.28398100
O	0.00017600	3.16006900	-0.11131500	O	3.24964500	-0.55046400	2.47760900
O	-0.09800100	2.20838700	-1.20812900	O	3.56403000	-1.03100400	1.15086200
H	-1.02014100	2.37337600	-1.50750000	H	4.59612000	-0.78176100	1.03852500
O	-2.68739000	2.79052900	-1.98279800	O	6.00770800	-0.54966100	1.11054600
H	-2.98479800	3.61630500	-1.58953300	H	6.26755200	0.16700600	1.69852900
H	-3.46260900	2.20001900	-1.99823400	H	6.40382400	-0.36424700	0.22062600
O	-4.96975600	1.13056500	-2.13288900	O	6.74113200	-0.05641100	-1.40990300
H	-5.31153100	0.94716700	-3.01389600	H	7.36425000	-0.59511900	-1.90828200
H	-5.05465100	0.30409200	-1.64042300	H	5.85742500	-0.27347000	-1.75920800

IM2(2w)				TS2(2w)			
C	-4.06342100	0.52420600	-0.62539400	C	3.76266200	0.09976700	-0.11496400
C	-2.78867700	0.89331400	-1.05395900	C	2.53234200	0.68613400	-0.40709600
C	-1.68691000	0.14118100	-0.68444800	C	1.38080100	-0.08316300	-0.36127400
C	-1.84998700	-0.98614200	0.11996000	C	1.46157000	-1.42780900	-0.01263900
C	-3.11153100	-1.35704800	0.55131500	C	2.67518200	-2.02305400	0.28207500
C	-4.22584400	-0.60490400	0.18026300	C	3.83747400	-1.25887400	0.22762900
C	0.46308700	-1.33759400	0.10099000	C	-0.86592200	-1.62967500	-0.17762100
C	0.62578900	-0.20573400	-0.70317900	C	-0.95247800	-0.25388200	-0.55031000
C	1.56527400	-2.07018700	0.52130600	C	-2.00739800	-2.43084500	-0.03357700
C	1.89492400	0.19355000	-1.08112300	C	-2.16511400	0.31493400	-0.77040800
C	2.83903400	-1.66374000	0.14612100	C	-3.24303800	-1.87605800	-0.25002000
C	3.00699300	-0.52437000	-0.64478100	C	-3.38787000	-0.46370000	-0.67007200
O	-0.77708200	-1.76439500	0.50454000	O	0.31390100	-2.20984700	0.05908000
O	-0.44774200	0.53525200	-1.13866900	O	0.18461800	0.50363100	-0.67109100
Cl	-5.79736400	-1.11117400	0.74433100	Cl	5.35592100	-2.02718200	0.59814200
Cl	-5.42218400	1.50095500	-1.11906700	Cl	5.18884500	1.09106800	-0.18394100
Cl	4.23332200	-2.57918600	0.67057000	Cl	-4.66321200	-2.80742100	-0.06881900
Cl	1.06903900	1.82969300	2.49372300	Cl	0.74302300	4.18868600	-0.00642800
H	-3.22203900	-2.23247400	1.17710300	H	2.71103200	-3.07004900	0.55142900
H	-2.64920100	1.76707200	-1.67616700	H	2.45252100	1.73705600	-0.65522200
H	2.02496500	1.07264700	-1.69910500	H	-2.24549200	1.36744700	-1.02145700
H	1.41611000	-2.94003800	1.14666300	H	-1.88374200	-3.46302100	0.26611200
H	5.35002700	-1.41448700	-1.72502500	H	-5.12047000	0.21066500	2.04580900
O	4.79669400	-0.73638700	-2.14601800	O	-4.40885400	0.38033100	1.41272100
O	4.26442200	-0.05100800	-0.94120200	O	-4.49089100	0.02757600	-0.89737600
H	4.62995200	1.80544800	-0.91434900	H	-3.98663200	1.91838000	1.48311700
O	4.66187000	2.76885200	-0.78307700	O	-3.65352500	2.87693800	1.50308100
H	5.59421900	3.00535000	-0.76152000	H	-3.37629900	3.05407900	2.40601600
H	3.55761400	3.43944600	0.45458800	H	-2.69911500	3.35062000	0.27997900
O	2.93659700	3.79104700	1.12733900	O	-2.13161300	3.57313400	-0.51616300
H	2.51194000	4.55993200	0.73475500	H	-2.59045600	4.27430600	-0.99011800
H	1.88812300	2.69013200	1.89489200	H	-0.55752700	3.91618400	-0.22026000
IM1(3w)				TS1(3w)			
C	-4.20027400	0.04410800	0.68956800	C	5.03506500	-0.73107700	0.19677100
C	-2.99567000	0.09122600	1.39059300	C	3.79448400	-1.36690400	0.21186600
C	-1.83268700	-0.36916000	0.80115200	C	2.63025400	-0.62811400	0.07831200
C	-1.86049800	-0.87968900	-0.49562900	C	2.69434400	0.75944800	-0.07120400
C	-3.05096700	-0.92694100	-1.19842400	C	3.92638500	1.39503000	-0.08639400
C	-4.22830000	-0.46629200	-0.60865400	C	5.10045100	0.65585200	0.04728400
C	0.44656000	-1.23280200	-0.40848100	C	0.35900000	0.84852400	-0.18922700
C	0.47447600	-0.72011800	0.89152300	C	0.29287000	-0.54036400	-0.03287100
C	1.62030000	-1.64150000	-1.01852700	C	-0.80884000	1.57033400	-0.33313700
C	1.67626800	-0.61979000	1.57111300	C	-0.92311200	-1.19496100	-0.01373500
C	2.83200000	-1.54397200	-0.33583300	C	-2.04756900	0.92095100	-0.32831400
C	2.85864700	-1.03308700	0.96012900	C	-2.11111100	-0.46006100	-0.10852400
O	-0.72345500	-1.35610600	-1.11230200	O	1.56672800	1.52927200	-0.20705800
O	-0.66649600	-0.31292100	1.53315800	O	1.43089100	-1.30506900	0.10090800
Cl	-5.70894600	-0.54714300	-1.53274800	Cl	6.62647900	1.50459300	0.02209500
Cl	-5.64400800	0.63669500	1.47423800	Cl	6.47392400	-1.70531300	0.36753900
Cl	4.29137100	-2.07408500	-1.15074600	Cl	-3.51272700	1.87718700	-0.47882600
Cl	4.35593500	-0.88595500	1.86369700	Cl	-3.49713200	-1.09179400	1.39350100
H	-3.05798800	-1.32328500	-2.20489300	H	3.96422000	2.46991000	-0.20229400
H	-2.95843000	0.49441000	2.39344200	H	3.72834600	-2.44057500	0.32710800
H	1.68554000	-0.21025700	2.57199900	H	-0.95624800	-2.26811900	0.11222100
H	1.58455500	-2.03126600	-2.02688500	H	-0.76252000	2.64579500	-0.44668900
H	-1.50708100	3.02535700	-1.48145300	H	-2.42215600	-0.07254000	-2.48337800

O	-1.23874400	2.76359600	-0.59058400	O	-2.78688900	-0.97483500	-2.56450100
O	-0.15021700	1.84691700	-0.88859800	O	-3.16996400	-1.26324400	-1.20197200
H	0.61665600	2.34773200	-0.52031300	H	-4.22346400	-1.02801400	-1.18339600
O	1.91546400	3.24368400	0.21606600	O	-5.59457100	-0.87677400	-1.41876000
H	1.65795300	4.07387800	0.62654300	H	-5.81812000	-0.33964400	-2.18528800
H	2.75463700	3.41286000	-0.26209600	H	-6.20702700	-0.60989000	-0.67782800
O	4.31924400	3.48100800	-1.10907200	O	-7.30724200	-0.13903800	0.45878900
H	4.91369900	4.22680900	-1.22629000	H	-7.87232800	-0.83315700	0.81130000
H	4.88110900	2.69645500	-0.94531200	H	-6.87143400	0.28382000	1.23262300
H	5.63836500	0.44931300	-1.24233700	H	-5.83460600	0.98588700	3.37824700
O	5.76654900	1.16594000	-0.60958100	O	-5.76303500	0.95416500	2.41967400
H	5.56913400	0.76744500	0.24821600	H	-4.92457400	0.49931200	2.22250800
IM2(3w)				TS2(3w)			
C	4.21118300	0.41816400	0.68592700	C	3.68679900	-0.32293600	-0.04653000
C	2.94021000	0.54999500	1.24459100	C	2.47524200	0.30608800	-0.33053300
C	1.87790500	-0.17375400	0.73087600	C	1.30694400	-0.43925100	-0.33317500
C	2.07668700	-1.03224800	-0.34985000	C	1.34988600	-1.79820400	-0.03749200
C	3.33393300	-1.16524600	-0.91227300	C	2.54558000	-2.43311900	0.24809000
C	4.40928200	-0.44280700	-0.39569100	C	3.72593200	-1.69539800	0.24069400
C	-0.20525500	-1.54803600	-0.36140700	C	-0.97938100	-1.94086000	-0.23078700
C	-0.40276300	-0.68499000	0.72029900	C	-1.03118400	-0.55316700	-0.56234300
C	-1.27886300	-2.21001000	-0.94319800	C	-2.13993000	-2.71633300	-0.11446700
C	-1.67899900	-0.47945900	1.21320800	C	-2.22987600	0.04350900	-0.78401400
C	-2.55925900	-2.00109500	-0.44817800	C	-3.36343900	-2.12355500	-0.30601200
C	-2.76314800	-1.12656800	0.62273300	C	-3.47235400	-0.69857000	-0.67594700
O	1.04249900	-1.77393000	-0.88612000	O	0.18587800	-2.55666200	-0.00754700
O	0.64335600	-0.02745700	1.32240900	O	0.12321200	0.18814400	-0.63515600
Cl	5.97844600	-0.64535400	-1.13212700	Cl	5.22044500	-2.51409900	0.59931800
Cl	5.52089400	1.34660400	1.36921500	Cl	5.13717700	0.63632500	-0.05397800
Cl	-3.91762500	-2.82883700	-1.17490900	Cl	-4.80572100	-3.02417400	-0.13840800
Cl	-0.97197800	2.49865000	-1.34264700	Cl	-0.33653900	5.93454800	0.11085900
H	3.47224700	-1.83307700	-1.75192700	H	2.55280400	-3.49041700	0.47631900
H	2.77179600	1.21720700	2.07918300	H	2.42543500	1.36952700	-0.53957600
H	-1.83750800	0.20126900	2.03982500	H	-2.29364000	1.10011500	-1.01387100
H	-1.10226000	-2.87128000	-1.78082600	H	-2.04248900	-3.76001800	0.15333200
H	-5.04389800	-2.40162700	1.46226100	H	-5.05938700	0.11444100	2.10692600
O	-4.48748600	-1.85206500	2.03783700	O	-4.35049400	0.15644900	1.44976400
O	-4.03451300	-0.83058300	1.05783700	O	-4.56402400	-0.15063300	-0.82668500
H	-4.36305100	0.90791400	1.74409600	H	-3.36702800	1.41681800	1.70002300
O	-4.29119800	1.84456600	1.99436200	O	-2.66673600	2.13020900	1.84747300
H	-4.99690300	2.02353600	2.62212400	H	-3.03017900	2.74194300	2.49401000
H	-3.53628900	3.01777500	0.87466300	H	-1.81764700	2.83517200	0.59497200
O	-3.06600400	3.62114800	0.26211400	O	-1.33725000	3.23682700	-0.17985100
H	-1.91988300	3.01012700	-0.45700400	H	-0.93439200	4.69638100	0.00078200
H	-3.65903100	3.80785100	-0.48872600	H	-0.47299000	2.80680700	-0.26504900
H	-4.01126900	4.53292000	-2.97977400	H	1.44321800	3.58406200	-1.89782800
O	-3.91203500	3.77855600	-2.39084100	O	1.51762200	3.37423000	-0.96027500
H	-3.06194500	3.37376000	-2.61894500	H	1.42416700	4.23321400	-0.51917700
P(0w)				P(1w)			
C	3.71461000	0.89289900	-0.00021700	C	3.77614200	0.85056000	0.02327400
C	2.42062300	1.40892800	-0.00057600	C	2.45149800	1.28093800	0.03808900
C	1.33320000	0.54929200	-0.00042900	C	1.42267100	0.35190100	0.02304400
C	1.53901200	-0.82862000	0.00008600	C	1.71814500	-1.00937800	-0.00690600
C	2.82130300	-1.35227800	0.00046100	C	3.03178000	-1.44825300	-0.02190600
C	3.91724700	-0.49367900	0.00030800	C	4.06897000	-0.51962900	-0.00675000
C	-0.77445000	-1.18392500	-0.00013500	C	-0.56753200	-1.51360200	-0.00850100
C	-0.99282600	0.22415200	-0.00046000	C	-0.87646300	-0.12279900	0.02321900

C	-1.85015200	-2.07363300	-0.00003900	C	-1.58398900	-2.47054300	-0.02766200
C	-2.25842200	0.72706300	-0.00050900	C	-2.17156200	0.29928300	0.03897200
C	-3.13711500	-1.58541100	-0.00018600	C	-2.89874000	-2.06458300	-0.01391600
C	-3.41443800	-0.14307200	-0.00028500	C	-3.26871700	-0.64315700	0.02289800
O	0.47552500	-1.70888700	0.00023700	O	0.71343600	-1.95661900	-0.02300200
O	0.07201100	1.09052400	-0.00082600	O	0.13015500	0.80904400	0.03851900
Cl	5.51661200	-1.18363900	0.00079000	Cl	5.71039900	-1.10285600	-0.02623500
Cl	5.05281600	2.00638000	-0.00042200	Cl	5.03880800	2.04914000	0.04302900
Cl	-4.47877200	-2.67010200	-0.00018400	Cl	-4.16853400	-3.23479200	-0.03758900
Cl	-5.13821000	3.32031200	0.00087700	Cl	-3.24103700	4.11206600	-0.15430400
H	2.95931400	-2.42522400	0.00085800	H	3.23988900	-2.50961400	-0.04531500
H	2.25227500	2.47755600	-0.00097600	H	2.21235800	2.33572000	0.06137300
H	-2.42149500	1.79751900	-0.00071200	H	-2.39428000	1.35977100	0.05857900
H	-1.64886200	-3.13690600	0.00016200	H	-1.31528600	-3.51844000	-0.05284900
O	-4.58205900	0.29170500	-0.00019300	O	-4.46283200	-0.28714500	0.03897800
H	-4.96816900	2.00987400	0.00076400	H	-5.20183000	1.32122300	0.18745900
				O	-5.54266200	2.23893900	0.26407600
				H	-6.31527100	2.29472700	-0.30663800
				H	-4.31738000	3.33795200	0.02237500
P(2w)				P(3w)			
C	-3.81013700	0.56458700	-0.51959500	C	4.03897900	-1.16169000	-0.05750300
C	-2.54657000	0.96428800	-0.94779600	C	2.65861000	-1.33823000	-0.11138100
C	-1.44120000	0.17536200	-0.67130900	C	1.81907500	-0.23600100	-0.05066200
C	-1.59840500	-1.01428500	0.03638900	C	2.36119500	1.04268900	0.06208000
C	-2.85005300	-1.42015300	0.46860700	C	3.73250800	1.22946800	0.11587200
C	-3.96394300	-0.63230900	0.19215600	C	4.57960600	0.12647900	0.05672300
C	0.70713200	-1.39407400	-0.07099600	C	0.20772000	1.95970100	0.06313700
C	0.87523300	-0.17277800	-0.78490900	C	-0.35154300	0.65334700	-0.04967600
C	1.80617000	-2.19405100	0.24618000	C	-0.61552800	3.08631900	0.11622300
C	2.11818600	0.24570800	-1.14776500	C	-1.70149900	0.47382400	-0.10543800
C	3.06945800	-1.78711000	-0.11624700	C	-1.98062400	2.92727300	0.05943900
C	3.29908400	-0.51454200	-0.81053100	C	-2.60238100	1.60093000	-0.05388200
O	-0.51656300	-1.82148000	0.32239600	O	1.54705900	2.15714000	0.12207100
O	-0.21291500	0.59063700	-1.11748600	O	0.46800600	-0.44354400	-0.10276900
Cl	-5.52503300	-1.17108600	0.74813800	Cl	6.30072000	0.39317400	0.12702100
Cl	-5.17306400	1.58441400	-0.88880400	Cl	5.06016800	-2.57002800	-0.13561500
Cl	4.44188600	-2.76003900	0.27416400	Cl	-3.01664300	4.30996800	0.12223000
Cl	1.14910200	1.87741600	2.26367400	Cl	-2.51439300	-3.09106800	-0.50105500
H	-2.95019900	-2.34579700	1.01927900	H	4.13276400	2.23059200	0.20324700
H	-2.41541000	1.88844800	-1.49420400	H	2.22626600	-2.32610100	-0.20085500
H	2.24492400	1.18234500	-1.67497700	H	-2.10345700	-0.53204400	-0.19511900
H	1.64172100	-3.11861000	0.78368900	H	-0.16129800	4.06477900	0.20101300
O	4.44974900	-0.11192800	-1.07537400	O	-3.84450000	1.48224800	-0.10184200
H	4.80742200	1.62055500	-1.10572500	H	-5.05709300	0.28769200	-0.17971600
O	4.89862300	2.58998200	-0.97340200	O	-5.83012300	-0.32201900	-0.26126800
H	5.84298300	2.76025300	-0.90703500	H	-6.48712400	0.13386200	-0.79543000
H	3.85227600	3.23437100	0.27542100	H	-5.54751500	-1.81895200	-0.45322100
O	3.23313200	3.60017800	0.94890900	O	-5.30594600	-2.80137000	-0.58384500
H	2.90187800	4.43034000	0.59330800	H	-4.23262500	-2.86903000	-0.72532800
H	2.08930400	2.62313700	1.67286700	H	-5.40492100	-3.29916300	0.27143100
				H	-4.73122600	-5.09356600	1.76655700
				O	-4.64450300	-4.15420400	1.57706800
				H	-3.73788800	-4.02351900	1.22863600
Nucleophilic substitution reaction of TCDD involving HO_2^{-}							
IM1(S1)				TS1(S1)			
C	-2.58194500	-1.27842900	-0.35587200	C	-2.61156100	-1.30527400	-0.35841300

C	-1.39054800	-0.56430500	-0.28516400	C	-1.42872900	-0.57815500	-0.33065300
C	-1.43114100	0.82164000	-0.03899100	C	-1.47322200	0.80741300	-0.09976100
C	-2.65396000	1.45685000	0.12339200	C	-2.69675700	1.43481300	0.09260000
C	-3.84679900	0.73646600	0.04281000	C	-3.88186800	0.70232700	0.05839500
C	-3.80857900	-0.63390200	-0.19949800	C	-3.83901800	-0.67282300	-0.16782500
H	-2.66901000	2.52260000	0.30915100	H	-2.71581900	2.50180100	0.27012500
C	0.95149400	-0.47135500	-0.21537700	C	0.91048400	-0.48854900	-0.43739700
C	2.17869700	-1.26518000	-0.14664800	C	2.11190400	-1.14189500	-0.57037600
C	3.36009300	-0.38752000	-0.29510200	C	3.31097000	-0.43175200	-0.43507000
C	3.28938400	0.98144200	-0.10888200	C	3.26480200	0.94480500	-0.22006200
C	2.08141000	1.65842700	0.07169100	C	2.03606500	1.60650800	-0.09036900
C	0.92002500	0.87993200	-0.04117500	C	0.86634900	0.89148900	-0.20735300
H	2.03271600	2.70469400	0.32944500	H	2.00000000	2.67118700	0.09797500
Cl	-5.27770300	-1.59330700	-0.31060300	Cl	-5.29925300	-1.64571500	-0.21822000
Cl	4.86323900	-1.19128600	-0.78954200	Cl	4.81044800	-1.24177300	-0.84287200
O	-0.29980800	1.57892900	0.01203500	O	-0.34581500	1.57146100	-0.07154800
O	-0.22515800	-1.22114100	-0.46055300	O	-0.26133100	-1.24018300	-0.54097100
H	-2.54048600	-2.34414700	-0.53729600	H	-2.56320500	-2.37257400	-0.52831600
H	2.17227100	-2.09815700	-0.86256700	H	2.13461200	-2.21129200	-0.71466700
Cl	-5.36351100	1.60108200	0.25089900	Cl	-5.39865100	1.55248400	0.30453900
Cl	4.76899900	1.96672100	-0.10594400	Cl	4.73173000	1.90275400	-0.03933100
O	2.33660100	-1.31568700	2.28875100	O	2.15188400	-1.15579100	2.42374300
H	3.14560000	-0.80563500	2.11388000	H	2.58137100	-0.45393900	2.92606800
O	2.26370400	-2.15439200	1.10708100	O	3.30449700	-1.72553400	1.69834600
		IM2(S1)				IM1(S2)	
C	-2.37615700	-1.44376900	0.07799300	C	-2.57136100	-1.27400300	-0.37163600
C	-1.17447400	-0.77747400	0.22653500	C	-1.37887400	-0.56591700	-0.25660700
C	-1.16200400	0.59932100	0.45752400	C	-1.42201300	0.81562200	0.01712500
C	-2.34993600	1.29914600	0.54607000	C	-2.64779300	1.45074900	0.16239400
C	-3.56569700	0.62925300	0.40538800	C	-3.83983700	0.73606400	0.04048200
C	-3.57870700	-0.74210400	0.17062200	C	-3.79928300	-0.62971500	-0.23047000
H	-2.32374400	2.36866200	0.70165800	H	-2.66457000	2.51303200	0.36720500
C	1.15856400	-0.78912400	0.16643100	C	0.96344400	-0.48078200	-0.12070300
C	2.34691000	-1.46538600	-0.02657900	C	2.20262500	-1.26549200	-0.11658500
C	3.55903200	-0.76994000	-0.02267200	C	3.36814000	-0.37900700	-0.31268100
C	3.55232900	0.60993700	0.19115500	C	3.29812700	0.97955400	-0.11985400
C	2.36211400	1.29419400	0.39499200	C	2.08771100	1.65809000	0.10542600
C	1.16591900	0.59220200	0.38120900	C	0.93143700	0.88194600	0.03336800
H	2.34389300	2.36597900	0.53497800	H	2.04950400	2.70584200	0.35894700
Cl	-5.07991700	-1.63489200	-0.00815400	Cl	-5.26779400	-1.58191200	-0.39544800
Cl	-0.05710300	0.59297300	-2.63095000	Cl	4.86215200	-1.18233000	-0.81282400
O	0.01405800	1.28113200	0.65643300	O	-0.29231700	1.57197100	0.10966500
O	-0.01334900	-1.50849700	0.18206400	O	-0.21282900	-1.22502900	-0.41063100
H	-2.37114200	-2.50558500	-0.12574300	H	-2.52849600	-2.33548000	-0.57650900
H	2.34405200	-2.53216700	-0.20626500	H	2.17110800	-2.08890500	-0.84309600
Cl	-5.04968300	1.55771400	0.53919600	Cl	-5.35878000	1.59955400	0.23427400
Cl	5.07376300	1.49836100	0.21263000	Cl	4.76334000	1.97953600	-0.18134500
O	5.43983500	-1.72108600	0.99516100	O	2.36201200	-1.34944100	2.31773300
H	6.03203400	-0.95316400	1.01075600	H	1.53931700	-0.83653100	2.24101700
O	4.71790100	-1.45178900	-0.28911900	O	2.30533500	-2.16913200	1.12219700
		TS1(S2)				IM2(S2)	
C	-2.62225100	-1.30791200	-0.35487900	C	-2.25706800	-0.75108100	0.00238000
C	-1.43641200	-0.58803000	-0.29127400	C	-1.00038000	-0.16691800	0.00360900
C	-1.47752300	0.79580000	-0.04641200	C	-0.85347100	1.21891300	0.02673600
C	-2.70271800	1.42664300	0.12652800	C	-1.96696200	2.03940800	0.05329400
C	-3.89049400	0.70068200	0.06154200	C	-3.23647200	1.46556500	0.05675400
C	-3.85038600	-0.67189800	-0.18046500	C	-3.38053500	0.07576700	0.03073500

H	-2.71999800	2.49159900	0.31604000	H	-1.84138600	3.11352300	0.07140300
C	0.90508500	-0.49971400	-0.39095200	C	1.32700800	-0.40311800	-0.05672200
C	2.10664100	-1.14593400	-0.56335200	C	2.45390200	-1.21280300	-0.10749000
C	3.30434100	-0.43248200	-0.44894700	C	3.72700400	-0.64567800	-0.16055000
C	3.25880800	0.94576400	-0.24194600	C	3.86182100	0.74601700	-0.14379100
C	2.03151500	1.59875100	-0.07780300	C	2.74200900	1.56413200	-0.07932500
C	0.86186500	0.87846100	-0.15825200	C	1.47727600	0.98932000	-0.03539500
H	1.99521200	2.66194300	0.11809300	H	2.83336300	2.64200500	-0.06905900
Cl	-5.31392100	-1.63687900	-0.27282400	Cl	-4.96377300	-0.66318200	0.03249600
Cl	4.79697300	-1.23940900	-0.88063500	Cl	-2.40811400	-4.08012700	-0.14189900
O	-0.34898500	1.55285200	0.01812000	O	0.39588400	1.82952300	0.02607800
O	-0.26706500	-1.25824200	-0.46888900	O	0.10393600	-1.00072100	-0.01792900
H	-2.57665500	-2.37290300	-0.53984300	H	-2.34622500	-1.85603400	-0.02623000
H	2.13110600	-2.21367200	-0.72209600	H	2.33759100	-2.28923300	-0.11604000
Cl	-5.40743200	1.55509200	0.28908800	Cl	-4.62313900	2.54538200	0.09350900
Cl	4.72324400	1.90971900	-0.11024000	Cl	5.45998600	1.47755600	-0.21930000
O	2.29685800	-1.04276700	2.48599500	O	5.50129200	-1.59897100	1.00818800
H	1.78972800	-1.83630100	2.69048500	H	6.21585600	-0.95453900	0.88722100
O	3.42782800	-1.61888700	1.72764600	O	4.81534900	-1.47064900	-0.30263900
Nucleophilic addition reaction of TCDD involving HO_2^-							
IM1(A1)				TS1(A1)			
C	2.34676800	-1.23058300	-0.09611600	C	2.44943100	-1.23742100	0.15837700
C	1.08774000	-0.63238000	0.34481700	C	1.18102700	-0.67366800	0.53446800
C	1.11449700	0.82867000	0.27631100	C	1.13429500	0.76853700	0.50349900
C	2.25186700	1.55800800	0.08445800	C	2.20670400	1.54433100	0.15500800
C	3.48777400	0.92618900	-0.14893900	C	3.44154400	0.96294300	-0.17834700
C	3.48388500	-0.46355800	-0.27028600	C	3.51912700	-0.42786300	-0.19130000
H	2.18596100	2.63974900	0.08973700	H	2.07855300	2.61906800	0.10831900
C	-1.20808500	-0.53379500	-0.27141500	C	-1.16297900	-0.53969400	-0.33494800
C	-2.40813100	-1.18230300	-0.54566500	C	-2.37829300	-1.11062000	-0.73900400
C	-3.62726800	-0.51776100	-0.42303500	C	-3.59231400	-0.45753300	-0.54982900
C	-3.65294000	0.80604500	0.00911200	C	-3.62685100	0.78158500	0.08508900
C	-2.45949500	1.46162700	0.30270300	C	-2.43521800	1.36165000	0.51540100
C	-1.23947900	0.80985300	0.15681800	C	-1.21905700	0.72644500	0.30597000
H	-2.46667800	2.49091300	0.63610200	H	-2.44344600	2.32542400	1.00785100
Cl	4.97366000	-1.32065900	-0.71700600	Cl	5.03128100	-1.23536200	-0.64148500
Cl	-5.10466400	-1.39132600	-0.81621800	Cl	-5.06694100	-1.23893400	-1.12351600
O	-0.10933100	1.50599400	0.43097300	O	-0.09890900	1.38119400	0.74152600
O	-0.04228400	-1.19184100	-0.45257200	O	-0.01530600	-1.16811300	-0.56310900
H	2.35421400	-2.28496300	-0.33362400	H	2.53887300	-2.31157600	0.08708200
H	-2.37658700	-2.21698500	-0.85988300	H	-2.34977100	-2.08245000	-1.21376400
Cl	4.94677600	1.91479800	-0.29242600	Cl	4.80683200	2.01325400	-0.57005600
Cl	-5.16308300	1.69280300	0.19291700	Cl	-5.13535500	1.65223700	0.35837700
O	0.65340400	-2.39485600	1.97086400	O	0.52531600	-2.59641300	1.79112900
H	1.60119500	-2.58274900	2.05544000	H	1.37253800	-2.78711400	2.22067900
O	0.69113300	-0.95073500	1.75774700	O	0.57513300	-1.14239600	1.75634500
IM1(A2)				TS1(A2)			
C	2.48157300	-1.37066400	0.31386300	C	-2.46281000	1.32340000	0.39602000
C	1.23182600	0.63472500	-0.23633300	C	-1.21697000	-0.64951600	-0.30789300
C	2.43327700	1.26879900	-0.54375600	C	-2.43667100	-1.24217800	-0.65598600
C	3.64892300	0.59497300	-0.44418700	C	-3.64476100	-0.56710000	-0.50596800
C	3.67473200	-0.72666400	-0.00595700	C	-3.66320400	0.71855900	0.02863200
H	2.40517400	2.30242200	-0.86205000	H	-2.42015100	-2.24912900	-1.05151200
C	-1.08047800	-0.74078200	0.27248100	C	1.09874900	0.71508200	0.35643900
C	-2.17414600	-1.47253700	-0.08631200	C	2.16449200	1.46817700	-0.05216600
C	-3.40950000	-0.84741400	-0.35152000	C	3.40293800	0.86517700	-0.34588000

C	-3.45866600	0.53799300	-0.26364100	C	3.49793200	-0.51450600	-0.23281500
C	-2.35771500	1.30482600	0.10432500	C	2.42431000	-1.29546600	0.19297800
C	-1.07691900	0.70631600	0.42597100	C	1.14853500	-0.71921200	0.49298400
H	-2.42496400	2.38461900	0.12215800	H	2.52547400	-2.37072200	0.26082600
Cl	5.18133500	-1.62452200	0.14998500	Cl	-5.16234500	1.61803700	0.24926600
Cl	-4.80494100	-1.84850800	-0.76757900	Cl	4.75440000	1.89011500	-0.83684400
O	0.07133600	1.31237900	-0.37429500	O	-0.07294300	-1.31142400	-0.49096400
O	0.13505300	-1.40335400	0.49274000	O	-0.12271200	1.34343700	0.58624900
H	2.48663400	-2.39896700	0.65049800	H	-2.45840900	2.32373500	0.80895500
H	-2.07780900	-2.54771300	-0.17931400	H	2.03856500	2.53773900	-0.16807500
Cl	5.12426300	1.45533800	-0.87402100	Cl	-5.12973300	-1.38133000	-0.99843100
Cl	-4.96628800	1.40536100	-0.60769400	Cl	5.01945600	-1.34765000	-0.58570400
C	1.26456100	-0.71045400	0.19226900	C	-1.25349100	0.66380900	0.22566300
O	-1.44273900	0.72449800	2.78615200	O	1.26485000	-0.85891700	2.84250800
H	-2.29886800	1.04951900	2.44854400	H	2.16293100	-1.15011800	2.60194100
O	-0.54355900	1.18460700	1.75105200	O	0.50668900	-1.28994500	1.67657500
IM1(A3)				TS1(A3)			
C	-2.47144800	1.36015300	0.31599700	C	-2.46004200	1.32423500	0.39023200
C	-1.22180700	-0.65899600	-0.19179300	C	-1.20871600	-0.67086400	-0.22769300
C	-2.42382800	-1.28316100	-0.52352000	C	-2.41867600	-1.26317400	-0.60348900
C	-3.63615000	-0.60031400	-0.45473300	C	-3.62713800	-0.57898300	-0.50363500
C	-3.66291000	0.72434500	-0.02650300	C	-3.65312100	0.71809500	0.00166500
H	-2.39691900	-2.31869100	-0.83566500	H	-2.39618300	-2.27853300	-0.97648700
C	1.09191300	0.71876300	0.32738700	C	1.10153200	0.69763500	0.39419500
C	2.17268100	1.46167400	-0.07729700	C	2.15515100	1.46044800	-0.04438000
C	3.39438400	0.84440800	-0.37191200	C	3.37916800	0.86330300	-0.37125100
C	3.44962800	-0.55058200	-0.27761200	C	3.46987700	-0.52717400	-0.26658800
C	2.36860600	-1.31588500	0.10992200	C	2.41341800	-1.30991100	0.16140000
C	1.10149800	-0.72369900	0.47706400	C	1.14586600	-0.73811700	0.52088100
H	2.46014600	-2.39017200	0.19050000	H	2.53324600	-2.37918000	0.26649000
Cl	-5.16532300	1.63549900	0.08871600	Cl	-5.15194200	1.63049400	0.15816900
Cl	4.77655500	1.84251900	-0.83874900	Cl	4.72455900	1.88508400	-0.88921000
O	-0.07088500	-1.35111400	-0.29990400	O	-0.06750600	-1.35643900	-0.36281700
O	-0.13187200	1.37585000	0.56983600	O	-0.12619300	1.32441600	0.67023700
H	-2.47606900	2.39125900	0.64439600	H	-2.46163000	2.33460100	0.77820500
H	2.06113200	2.53445700	-0.18265300	H	2.02073400	2.53017000	-0.15193600
Cl	-5.10793800	-1.45317100	-0.91127200	Cl	-5.10075700	-1.39606300	-1.02110500
Cl	4.95913700	-1.40459600	-0.64602800	Cl	4.99023300	-1.35142400	-0.65192400
C	-1.25727600	0.69004900	0.22909800	C	-1.24967000	0.65415000	0.27375400
O	1.46467300	-0.72143400	2.84946800	O	1.38165200	-0.84955000	2.88620700
H	1.43395800	0.24375300	2.73687800	H	1.26636100	0.11467500	2.87851700
O	0.56616800	-1.17897600	1.81242600	O	0.54904800	-1.26307100	1.77185400
IM2(A2)				TS2(A2)			
C	-2.26644700	0.97315200	0.74144600	C	-2.29759400	-1.08364500	0.32115100
C	-1.29606800	-0.07493800	-1.30752900	C	-1.29253700	1.20137300	0.26333000
C	-2.63798400	-0.52533800	-1.57358200	C	-2.65318400	1.69544200	0.22670700
C	-3.70902300	-0.23257300	-0.74721400	C	-3.73482100	0.85760500	0.17476200
C	-3.55181300	0.52177200	0.41877800	C	-3.56804800	-0.55132700	0.21031600
H	-2.79437100	-1.10327100	-2.47547200	H	-2.78038800	2.76862400	0.17637200
C	1.19803900	0.57067800	0.20325400	C	1.17609400	-0.35010000	0.29385600
C	2.33053000	1.31752100	-0.12358000	C	2.24878500	-0.49428100	1.17170400
C	3.59358700	0.74184300	-0.14887600	C	3.52693500	-0.16495400	0.76585100
C	3.75591700	-0.60761300	0.16735500	C	3.76204800	0.24723800	-0.57485400
C	2.64014700	-1.35067100	0.53287500	C	2.74000000	0.29865700	-1.48155700
C	1.37023600	-0.78446900	0.55643300	C	1.37441600	0.01690100	-1.10460200
H	2.73853900	-2.39316600	0.80476000	H	2.92012500	0.58738200	-2.50821500
Cl	-4.89554200	0.93622300	1.48644900	Cl	-4.94091800	-1.63584300	0.10473200

Cl	4.96279200	1.74736400	-0.59766700	Cl	4.83428400	-0.26711700	1.92772200
O	-0.30868000	-0.29219800	-2.05467900	O	-0.29916900	1.95713200	0.23792300
O	0.03324700	1.26306400	0.26626800	O	-0.00852300	-0.83537600	0.68556400
H	-2.11209200	1.58961300	1.61963800	H	-2.12029900	-2.15095700	0.30001500
H	2.19727500	2.35532500	-0.39689100	H	2.06295100	-0.83688400	2.18067300
Cl	-5.31249000	-0.83868300	-1.20684400	Cl	-5.34929800	1.56052400	0.05916800
Cl	5.33091200	-1.38799000	0.14499200	Cl	5.38918200	0.67664000	-1.09588200
C	-1.19006400	0.67320200	-0.07341800	C	-1.19020000	-0.24638300	0.36143400
O	-0.28373100	-1.19694100	2.13874900	O	-0.04882800	-2.66166900	-1.33784900
H	-1.08439800	-0.75855800	1.79056500	H	-0.07925700	-2.38246200	-2.26179400
O	0.33120000	-1.62303300	0.86515400	O	0.42905300	0.01162500	-1.90854000
Transition states in the substitution processes							
F-TCDD + H_2O_2				Br-TCDD + H_2O_2			
C	5.14130800	1.01184500	0.21529300	C	-4.67228600	-0.71679600	0.02855300
C	3.85393500	1.48106600	0.42276400	C	-3.43688400	-1.35365300	0.15339900
C	2.77288300	0.64915000	0.16050500	C	-2.26495000	-0.61515900	0.13667800
C	2.98253400	-0.65141100	-0.30808600	C	-2.31476800	0.77332100	-0.00661500
C	4.27735200	-1.11472000	-0.51252800	C	-3.53975900	1.41006000	-0.13164400
C	5.35103300	-0.27906200	-0.24967800	C	-4.72309000	0.67131800	-0.11463800
C	0.66925000	-0.99958600	-0.33947000	C	0.02142700	0.86247700	0.09380600
C	0.45600300	0.29726500	0.12500100	C	0.07301900	-0.52951100	0.23290600
C	-0.42352400	-1.82269900	-0.56023800	C	1.19574200	1.58793700	0.08320200
C	-0.82100100	0.77814300	0.36832100	C	1.28318500	-1.18432900	0.34767100
C	-1.69659900	-1.34035600	-0.31295000	C	2.43060400	0.94059200	0.21237200
C	-1.94495200	-0.02024400	0.10360600	C	2.47522300	-0.45391000	0.27868200
O	1.94358100	-1.50348300	-0.58179700	O	-1.17884500	1.54318100	-0.02840300
O	1.50982500	1.15526700	0.37665100	O	-1.07275100	-1.29292500	0.26403700
H	4.44423100	-2.12037500	-0.87566900	H	-3.56061000	2.48588400	-0.24217700
H	3.69078000	2.48757000	0.78526500	H	-3.37648800	-2.42800900	0.26465500
H	-0.95480000	1.78549200	0.73842700	H	1.30367100	-2.26015800	0.45144800
H	-0.28893200	-2.83227100	-0.92753600	H	1.15300000	2.66373300	-0.02720400
H	-2.07613900	-1.30300800	2.07813400	H	2.88737000	0.42350600	2.58274300
O	-2.64836500	-0.59951200	2.44249700	O	3.14854700	-0.48356000	2.82903100
O	-3.08762100	0.08394900	1.25510900	O	3.53172100	-1.04342700	1.55211200
H	-4.11460000	-0.43426400	0.92171000	H	4.58087700	-0.86169700	1.51112500
O	-5.15187800	-0.96744800	0.58261400	O	5.98134700	-0.72025200	1.79767800
H	-4.93307000	-1.56131700	-0.14858900	H	6.20670700	-0.07846300	2.47791800
H	-5.82655000	-0.28903300	0.25094700	H	6.66316400	-0.64130700	1.07740000
O	-6.78497600	0.80801700	-0.33012800	O	7.88355000	-0.45706100	-0.04201200
H	-7.48668000	1.20771700	0.19144900	H	8.40765300	-1.24298500	-0.22387200
H	-6.26325500	1.53503100	-0.74933700	H	7.52967200	-0.15499700	-0.90746500
H	-4.78320200	3.38519400	-1.40344800	H	6.67551900	0.26757600	-3.22183500
O	-5.00396500	2.45218500	-1.46868800	O	6.53405200	0.34848800	-2.27392900
H	-4.18051100	1.95227700	-1.30268800	H	5.65984000	-0.04116500	-2.09229500
F	-2.78270800	-2.15217200	-0.55735100	Br	4.02479100	1.99618000	0.16734200
F	-3.03815100	0.70800400	-0.84404300	Br	3.95166700	-1.52180600	-1.14788600
F	6.19614700	1.81153600	0.46550000	Br	-6.23813800	-1.80401700	0.06274100
F	6.60810900	-0.72083000	-0.44676700	Br	-6.36449500	1.62559800	-0.29147700
TCDD + CH_3OOH				F-TCDD + HO_2^- (substitution reaction)			
C	-5.02254000	-1.01388800	0.13773600	C	-2.64032300	-1.27395200	-0.53491500
C	-3.71219400	-1.48826400	0.17805300	C	-1.48318500	-0.50381000	-0.47216100
C	-2.64802100	-0.61312900	0.03371400	C	-1.55610700	0.82716000	-0.02980300
C	-2.88446700	0.75118300	-0.15466700	C	-2.78207500	1.36961600	0.34164700
C	-4.18628600	1.22607700	-0.19657600	C	-3.92845300	0.59349800	0.26557600
C	-5.25996100	0.34926200	-0.05007800	C	-3.85775400	-0.71967500	-0.16999100
C	-0.57876200	1.12623200	-0.28124500	H	-2.83774700	2.39412400	0.68591900

C	-0.34126700	-0.23648200	-0.09464500	C	0.84791500	-0.32426900	-0.63802100
C	0.49021700	1.98709700	-0.45047400	C	2.06484000	-0.93672800	-0.85095900
C	0.94976700	-0.73451400	-0.08146200	C	3.23375000	-0.22694400	-0.59816700
C	1.79751600	1.50022000	-0.43531600	C	3.15862100	1.09130900	-0.17592300
C	2.02034800	0.13148800	-0.29025500	C	1.93287800	1.71234800	0.04405800
O	-1.86121700	1.65350400	-0.30063000	C	0.77526100	0.99804800	-0.19576000
O	-1.37392700	-1.13304800	0.08470600	H	1.89020500	2.73489200	0.39588200
Cl	-6.88004700	0.99873900	-0.10877600	O	-0.45236600	1.62793600	0.03208400
Cl	-6.32946400	-2.15678500	0.32621500	O	-0.31107700	-1.07136400	-0.86743300
Cl	3.12307900	2.62524000	-0.65765500	H	-2.58409900	-2.30283400	-0.86554800
Cl	3.41170000	-0.63185700	-1.77098600	H	2.12257700	-1.97703800	-1.13381000
H	-4.35729100	2.28407000	-0.34402800	O	2.08659100	-1.43784700	2.10932200
H	-3.51341200	-2.54184700	0.32212900	H	2.50654500	-0.86628500	2.76119600
H	1.11442200	-1.79301300	0.06164300	O	3.27137800	-1.86506200	1.31755800
H	0.30649800	3.04353600	-0.59792600	F	-4.98710700	-1.46696600	-0.23794000
O	2.96257500	-0.22280000	2.24459100	F	-5.12593800	1.12313200	0.61984300
O	3.30366100	-0.34120500	0.84636000	F	4.30185700	1.79242700	0.06050800
H	4.28101300	0.05970100	0.71048100	F	4.42831000	-0.74323400	-0.94123200
O	5.63413000	0.53825700	0.59829700				
H	5.69777000	0.98824400	-0.25278200				
H	6.26433500	-0.23120900	0.56235500				
O	7.08327600	-1.68410900	0.47562000				
H	7.98650300	-1.81207200	0.17283300				
H	6.51532900	-2.31601700	-0.02242400				
H	4.76058600	-3.85562600	-0.56777700				
O	5.27705200	-3.13336400	-0.93841900				
H	4.62874300	-2.46670200	-1.23533800				
C	3.08533500	1.13021300	2.70682300				
H	2.34636300	1.78047600	2.23818300				
H	2.87905300	1.04078700	3.77532000				
H	4.09802400	1.50809400	2.55391600				
Br-TCDD + HO_2^- (substitution reaction)				TCDD + $CH_3O_2^-$ (substitution reaction)			
C	-2.54031500	-1.32979200	-0.22908100	C	-2.76233600	-1.27922300	-0.49977300
C	-1.34607000	-0.62089000	-0.24551600	C	-1.58196200	-0.54857800	-0.47444600
C	-1.37222800	0.78085700	-0.15348600	C	-1.62038900	0.81931700	-0.15738500
C	-2.58890000	1.44182100	-0.05090000	C	-2.83640500	1.42675400	0.12520500
C	-3.78677800	0.72722800	-0.04045700	C	-4.01984500	0.69129800	0.09383400
C	-3.76232100	-0.66411400	-0.12984400	C	-3.98279100	-0.66642800	-0.21975500
H	-2.58845200	2.52125600	0.01956100	H	-2.85093000	2.48029200	0.37041300
C	0.99567700	-0.57638800	-0.30444800	C	0.75114600	-0.44077700	-0.66713100
C	2.18867500	-1.25710500	-0.34189800	C	1.94939600	-1.08002700	-0.87839300
C	3.39678700	-0.55318800	-0.24992300	C	3.15209600	-0.37507200	-0.74476800
C	3.36989200	0.83667600	-0.17251700	C	3.10844500	0.98792300	-0.45208900
C	2.14759400	1.52708400	-0.13782500	C	1.88638000	1.63096200	-0.23373200
C	0.97022300	0.82062600	-0.21261500	C	0.71220300	0.91921500	-0.35050100
H	2.12034000	2.60533300	-0.05719800	H	1.85462700	2.68064200	0.02597300
O	-0.23300300	1.52733000	-0.17566200	Cl	-5.44088800	-1.64159800	-0.27279100
O	-0.18598700	-1.31668800	-0.36304000	Cl	4.63247200	-1.15048000	-1.26966600
H	-2.50071200	-2.40894200	-0.29200400	O	-0.49504200	1.58598700	-0.12918900
H	2.19206000	-2.33554300	-0.38161900	O	-0.42122800	-1.19079500	-0.77452900
O	2.12505000	-0.99668900	2.62747500	H	-2.71816600	-2.33336300	-0.73890800
H	2.56934800	-0.26793300	3.07589600	H	1.96848600	-2.14015900	-1.08195000
O	3.26990400	-1.67575600	1.99445800	Cl	-5.52698000	1.51589900	0.45543800
Br	-5.35549300	-1.72634700	-0.12104100	Cl	4.57633900	1.94064500	-0.28023500
Br	-5.41551800	1.72602500	0.09685800	O	2.24203700	-1.17415900	2.16602800
Br	5.01499800	-1.52844200	-0.56710200	O	3.33398000	-1.71276600	1.36144200
Br	4.96647800	1.89400300	-0.02069900	C	2.82205200	-0.56274600	3.27896200

H	3.38030300	-1.28957800	3.89251200
H	3.51887300	0.23879200	2.98597300
H	2.00486100	-0.13438200	3.87914900

F-TCDD + HO_2^- (addition reaction)

C	-2.50422400	1.31209700	0.49038700
C	-1.25574500	-0.55396600	-0.46204800
C	-2.47320200	-1.12126000	-0.85860600
C	-3.67271800	-0.47623600	-0.60329500
C	-3.69148700	0.73077200	0.07314900
H	-2.46899000	-2.07573000	-1.36924100
C	1.06314600	0.74225900	0.29434600
C	2.11310100	1.55659200	-0.04194800
C	3.33202300	1.00005500	-0.44874500
C	3.43776500	-0.37162500	-0.52382600
C	2.39148900	-1.22141400	-0.17926500
C	1.11641600	-0.70255800	0.23170700
H	2.51226000	-2.29229400	-0.28533600
O	-0.10443500	-1.18225200	-0.74365300
O	-0.15736700	1.32389500	0.64533300
H	-2.51715200	2.25747500	1.01781600
H	1.99096400	2.63295300	-0.00940600
C	-1.29023300	0.68678300	0.22208800
O	1.37277500	-1.15714500	2.54072500
H	2.25543100	-1.36386300	2.18017200
O	0.53512300	-1.42345100	1.38207800
F	4.39566100	1.81602600	-0.75494100
F	4.62283800	-0.92737600	-0.91860700
F	-4.84513800	-1.03602800	-1.01526100
F	-4.87772400	1.35449800	0.32286200

Br-TCDD + HO_2^- (addition reaction)

C	-2.42655400	1.30913600	0.42928800
C	-1.17805600	-0.71456700	-0.10625700
C	-2.39595600	-1.32735900	-0.42861000
C	-3.60388000	-0.63869300	-0.34934300
C	-3.62545100	0.68344600	0.08812700
H	-2.37243800	-2.36069100	-0.74785500
C	1.13236000	0.69000700	0.49461800
C	2.20691300	1.40542500	0.04369100
C	3.44970800	0.77830300	-0.18191100
C	3.53649100	-0.58904500	0.04218600
C	2.45319700	-1.33069500	0.51113600
C	1.17600900	-0.72780100	0.74460100
H	2.54381700	-2.39695600	0.67053200
O	-0.03593500	-1.39124600	-0.22122200
O	-0.08983700	1.33659500	0.65416900
H	-2.41876800	2.33664500	0.76848100
H	2.08226100	2.46215100	-0.15785300
C	-1.21770900	0.63482300	0.32744400
O	1.23746200	-0.68301200	3.09734600
H	2.13701200	-1.00877100	2.91530100
O	0.50813300	-1.20594300	1.95050800
Br	4.91679300	1.86703000	-0.77641900
Br	5.18664900	-1.55240300	-0.23267900
Br	-5.20265200	-1.58083600	-0.85556000
Br	-5.24287300	1.70845400	0.23651900

TCDD + $CH_3O_2^-$ (addition reaction)

C	-2.57103100	1.20861400	0.73700800
C	-1.31421300	-0.41801900	-0.55877900
C	-2.52239600	-0.90637900	-1.05732200
C	-3.74048000	-0.34150500	-0.68571400
C	-3.76726400	0.71531000	0.22034700
H	-2.49489200	-1.74136100	-1.74485200
C	0.99767600	0.71733100	0.40417400
C	2.06257500	1.56942300	0.30972900
C	3.29636600	1.12519200	-0.19593200
C	3.37262700	-0.19452300	-0.63330700
C	2.30103200	-1.07034400	-0.55167300
C	1.03738700	-0.67600100	0.01778000
H	2.39895400	-2.08523800	-0.91204100
Cl	-5.27724900	1.46387800	0.73373600
Cl	4.65950300	2.24936900	-0.25144100
O	-0.15742300	-0.97128200	-0.96442200
O	-0.22470700	1.21245900	0.88035200
H	-2.57603200	2.03261400	1.43839200
H	1.93775800	2.60051900	0.61783700
Cl	-5.21990900	-1.00077000	-1.38102200
Cl	4.88698800	-0.82978300	-1.30586300
C	-1.35166400	0.66467200	0.35171200
O	1.26748400	-1.45681600	2.26800800
O	0.49046800	-1.62094100	1.05064100
C	2.05186700	-2.62490400	2.42787900
H	1.42149600	-3.52104300	2.46873800
H	2.79355200	-2.72302000	1.62982000

HO_2^-

O	0.05584900	-0.70724200	0.00000000
H	-0.89359000	-0.86354200	0.00000000
O	0.05584900	0.81518500	0.00000000

H 2.55903000 -2.48885100 3.38805600	
F-TCDD	Br-TCDD
C -2.36984200 -1.39721500 0.00000400<br>C -1.17075200 -0.69794100 0.00004800<br>C -1.17075200 0.69794400 0.00004400<br>C -2.36984200 1.39721100 -0.00000500<br>C -3.56474300 0.69417500 -0.00004500<br>C -3.56474400 -0.69417900 -0.00004100<br>H -2.37237800 2.47933800 -0.00000900<br>C 1.17075200 -0.69794000 0.00005800<br>C 2.36984200 -1.39721500 0.00002300<br>C 3.56474400 -0.69417900 -0.00001100<br>C 3.56474300 0.69417500 -0.00001600<br>C 2.36984200 1.39721100 0.00001500<br>C 1.17075200 0.69794400 0.00005300<br>H 2.37237800 2.47933800 0.00001100<br>O 0.00000000 1.42652300 0.00009200<br>O 0.00000000 -1.42652100 0.00010400<br>H -2.37237100 -2.47933900 0.00000700<br>H 2.37237100 -2.47933900 0.00002600<br>F -4.73322000 1.35947200 -0.00008900<br>F -4.73321600 -1.35947000 -0.00008100<br>F 4.73321600 -1.35947000 -0.00004200<br>F 4.73322000 1.35947200 -0.00005000	C -2.36910400 -1.39054000 0.00004300<br>C -1.16988700 -0.69781500 0.00009200<br>C -1.16985700 0.69770200 0.00008700<br>C -2.36906700 1.39057800 0.00003700<br>C -3.58100500 0.69828000 -0.00000600<br>C -3.58100600 -0.69823200 -0.00000500<br>H -2.34918900 2.47213400 0.00003500<br>C 1.16988700 -0.69781400 0.00010200<br>C 2.36910400 -1.39054000 0.00006300<br>C 3.58100600 -0.69823100 0.00002500<br>C 3.58100400 0.69828000 0.00002300<br>C 2.36906700 1.39057800 0.00005600<br>C 1.16985600 0.69770300 0.00009700<br>H 2.34919000 2.47213500 0.00005300<br>O 0.00000000 1.42538200 0.00013500<br>O 0.00000000 -1.42547900 0.00015700<br>H -2.34936700 -2.47214400 0.00004600<br>H 2.34936700 -2.47214400 0.00006600<br>Br -5.18679100 1.72441200 -0.00006500<br>Br -5.18680700 -1.72439700 -0.00006400<br>Br 5.18680700 -1.72439700 -0.00002400<br>Br 5.18679100 1.72441200 -0.00002500
CH_3OOH	CH_3OO^-
O -0.01605300 0.60355700 -0.03870900<br>O -1.15832800 -0.30500300 -0.07093800<br>H -1.73721500 0.13023900 0.56997700<br>C 1.13533400 -0.21903000 0.02961600<br>H 1.19147000 -0.88847800 -0.83413100<br>H 1.97166400 0.48334900 0.00599300<br>H 1.15713000 -0.79935700 0.95764600	O -0.05767800 0.60813900 -0.00000400<br>O -1.24362400 -0.27552900 0.00001400<br>C 1.05121500 -0.22061000 -0.00002500<br>H 1.07514400 -0.87761200 0.89140800<br>H 1.07441400 -0.87841400 -0.89084600<br>H 1.95356500 0.41880800 -0.00049000

* All the geometries have been obtained at the B3LYP/6-311++G** level of theory.

Table S3. Calculated energy parameters for the optimized species in the present study*

TCDD	H_2O_2	TS1	TS2	TS3	TS4
-2451.030752<br>-2451.015921<br>-2451.014977<br>-2451.073408	-151.575731<br>-151.572488<br>-151.571544<br>-151.597391	-2602.523461<br>-2602.505439<br>-2602.504494<br>-2602.571303	-2602.523204<br>-2602.504944<br>-2602.504000<br>-2602.571379	-2602.520460<br>-2602.502244<br>-2602.501300<br>-2602.568741	-2602.523784<br>-2602.505408<br>-2602.504464<br>-2602.572581
IM1(0w)	TS1(0w)	IM2(0w)	TS2(0w)	IM1(1w)	TS1(1w)
-2602.608905<br>-2602.588789<br>-2602.587844<br>-2602.664004	-2602.523461<br>-2602.505439<br>-2602.504494<br>-2602.571303	-2602.618952<br>-2602.599592<br>-2602.598648<br>-2602.671328	-2602.611157<br>-2602.592829<br>-2602.591884<br>-2602.661339	-2679.054612<br>-2679.031370<br>-2679.030426<br>-2679.113051	-2678.977198<br>-2678.956167<br>-2678.955223<br>-2679.028582
IM2(1w)	TS2(1w)	IM1(2w)	TS1(2w)	IM2(2w)	TS2(2w)
-2679.065619<br>-2679.043332<br>-2679.042388<br>-2679.122782	-2679.056346<br>-2679.035493<br>-2679.034549<br>-2679.109189	-2755.502992<br>-2755.476947<br>-2755.476003<br>-2755.564194	-2755.430219<br>-2755.406699<br>-2755.405755<br>-2755.484852	-2755.513329<br>-2755.487971<br>-2755.487026<br>-2755.576259	-2755.495374<br>-2755.471110<br>-2755.470166<br>-2755.553807
IM1(3w)	TS1(3w)	IM2(3w)	TS2(3w)	P(0w)	P(1w)
-2831.952161<br>-2831.922913<br>-2831.921969<br>-2832.019108	-2831.880399<br>-2831.853788<br>-2831.852844<br>-2831.939970	-2831.959308<br>-2831.931092<br>-2831.930148<br>-2832.026082	-2831.937352<br>-2831.909991<br>-2831.909047<br>-2831.999999	-2526.856398<br>-2526.839163<br>-2526.838219<br>-2526.905889	-2603.304846<br>-2603.284663<br>-2603.283719<br>-2603.358911
P(2w)	P(3w)	IM1(S1)	TS1(S1)	IM2(S1)	IM1(S2)
-2679.752338<br>-2679.729136<br>-2679.728192<br>-2679.811530	-2756.198450<br>-2756.173577<br>-2756.172633<br>-2756.259604	-2602.051809<br>-2602.033504<br>-2602.032560<br>-2602.100057	-2602.038930<br>-2602.020375<br>-2602.019431<br>-2602.087879	-2602.111094<br>-2602.092254<br>-2602.091310<br>-2602.160921	-2602.050229<br>-2602.031926<br>-2602.030982<br>-2602.098509
TS1(S2)	IM2(S2)	IM1(A1)	TS1(A1)	IM1(A2)	TS1(A2)
-2602.037827<br>-2602.019128<br>-2602.018183<br>-2602.087225	-2602.110862<br>-2602.092096<br>-2602.091152<br>-2602.162103	-2602.058776<br>-2602.040555<br>-2602.039611<br>-2602.106580	-2602.057830<br>-2602.040018<br>-2602.039074<br>-2602.105056	-2602.062825<br>-2602.044923<br>-2602.043979<br>-2602.109976	-2602.062960<br>-2602.045498<br>-2602.044554<br>-2602.109510
IM1(A3)	TS1(A3)	IM2(A2)	TS2(A2)		
-2602.061066<br>-2602.042866<br>-2602.041922<br>-2602.108777	-2602.061459<br>-2602.043826<br>-2602.042882<br>-2602.108311	-2602.098038<br>-2602.079718<br>-2602.078774<br>-2602.146416	-2602.073734<br>-2602.054586<br>-2602.053642<br>-2602.123850		
Transition states in the substitution processes					
F-TCDD + H_2O_2	Br-TCDD + H_2O_2	TCDD + CH_3OOH	F-TCDD + HO_2^- (substitution reaction)	Br-TCDD + HO_2^- (substitution reaction)	TCDD + $CH_3O_2^-$ (substitution reaction)
-1390.454759<br>-1390.430285<br>-1390.429340<br>-1390.510873	-11287.564559<br>-11287.536511<br>-11287.535566<br>-11287.628780	-2871.165957<br>-2871.138027<br>-2871.137083<br>-2871.227404	-1160.609479<br>-1160.592399<br>-1160.591454<br>-1160.655594	-11057.720557<br>-11057.700674<br>-11057.699730<br>-11057.773962	-2641.328978<br>-2641.309133<br>-2641.308189<br>-2641.380893

F-TCDD + HO_2^- (addition reaction)	Br-TCDD + HO_2^- (addition reaction)	TCDD + $CH_3O_2^-$ (addition reaction)	HO_2^-	F-TCDD	Br-TCDD
-1160.626895<br>-1160.611055<br>-1160.610110<br>-1160.669890	-11057.745592<br>-11057.726904<br>-11057.725959<br>-11057.796353	-2641.346489<br>-2641.327728<br>-2641.326784<br>-2641.394665	-150.982553<br>-150.979609<br>-150.978665<br>-151.004354	-1009.602212<br>-1009.588853<br>-1009.587909<br>-1009.643221	-10906.711682<br>-10906.695598<br>-10906.694654<br>-10906.759919
CH_3OOH	CH_3OO^-	H_2O			
-190.864844<br>-190.860435<br>-190.859491<br>-190.890460	-190.277536<br>-190.273792<br>-190.272848<br>-190.302351	-76.437242<br>-76.434406<br>-76.433462<br>-76.454884			

* The energy parameters from top to bottom refer to the sums of electronic and zero-point energies, electronic and thermal energies, electronic and thermal enthalpies, electronic and thermal free energies, respectively. All the results have been obtained at the B3LYP/6-311++G** level of theory.
